# Introduction of a 4-Hexyl-2-thienyl Substituent on Pyridine Rings as a Route for Brightly Luminescent 1,3-Di-(2-pyridyl)benzene Platinum(II) Complexes

**DOI:** 10.3390/molecules30224410

**Published:** 2025-11-14

**Authors:** Alessia Colombo, Claudia Dragonetti, Francesco Fagnani, Dominique Roberto, Simona Fantacci, Daniele Marinotto

**Affiliations:** 1 UdR INSTM di Milano, Dipartimento di Chimica, Università degli Studi di Milano, via C. Golgi 19, 20133 Milan, Italy; alessia.colombo@unimi.it (A.C.); claudia.dragonetti@unimi.it (C.D.); 2 Computational Laboratory for Hybrid/Organic Photovoltaics (CLHYO), Istituto di Scienze e Tecnologie Chimiche “Giulio Natta” SCITEC, Consiglio Nazionale delle Ricerche (CNR), via Elce di Sotto 8, 06213 Perugia, Italy; simona.fantacci@cnr.it; 3 Istituto di Scienze e Tecnologie Chimiche (SCITEC) “Giulio Natta”, Consiglio Nazionale delle Ricerche (CNR), via C. Golgi 19, 20133 Milan, Italy; daniele.marinotto@scitec.cnr.it

**Keywords:** platinum(II) complexes, *N^C^N* ligand, luminescence

## Abstract

The synthesis and characterization of two new complexes, namely Pt(1,3-bis(4-(4-hexyl-2-thienyl)-pyridin-2-yl)-5-mesitylbenzene)Cl and Pt(1,3-bis(4-(4-hexyl-2-thienyl)-pyridin-2-yl)-5-(2-thienyl)benzene)Cl, are reported. Both exhibit luminescence quantum yields approaching unity (Φlum = 0.96–0.99) in the green region of the visible spectrum (534–554 nm) in diluted degassed dichloromethane solution. Similarly to other *N^C^N* platinum(II) complexes, a broad emission band grows in the deep red region (738–752 nm) upon increasing the concentration, due to the creation of bi-molecular emissive excited states. Interestingly, it appears that the introduction of a 2-thienyl group on the pyridine rings is a route to maintain excellent quantum yields even in concentrated solution. In order to have an insight into the electronic properties of the novel compounds, density functional theory (DFT) and time-dependent (TD)DFT approaches were employed to calculate the molecular geometry, the ground state, the electronic structure and the excited electronic states of the complexes, both as a monomers and dimers in solution.

## 1. Introduction

Today, a lot of research work is dedicated to platinum(II) complexes, due to their appealing properties in various fields of optoelectronics and photonics, like photocatalysis [1,2,3], nonlinear optics [4,5,6,7,8,9,10,11,12], sensing [13,14,15,16,17,18,19,20,21,22,23,24,25,26,27], Organic Light Emitting Diodes [28,29,30,31,32,33,34,35,36,37,38,39,40,41,42,43,44,45,46,47,48,49,50,51,52,53,54,55,56], and biomedicine [57,58,59,60,61,62,63,64,65,66,67,68,69,70,71]. The platinum atom is associated with a strong spin–orbit coupling, promoting intersystem crossing to triplet excited states and their subsequent radiative decay with emission of light [72,73]. In addition, platinum(II) complexes are characterized by a square planar geometry which can allow Pt–Pt or ligand–ligand intermolecular interactions, leading to bi-molecular states in the excited (excimers) or in the ground (dimers) states. This aspect is of particular importance because the parallel emissions from mono-molecular and bi-molecular excited states are an efficient way to modulate the color of OLEDs by choosing the suitable quantity of the platinum complex in the host matrix used for the fabrication of the emissive layer [74].

Thus, a huge amount of square planar platinum(II) complexes has been designed. In particular, twenty-five years ago, Cardenas et al. reported that K_2_PtCl_4_ reacts with 1,3-bis(pyridin-2-yl)benzene (bpybH) in acetic acid, generating Pt(bpyb)Cl, the first Pt(II) compound with a cyclometalated terdentate *N^C^N* 1,3-bis(pyridin-2-yl)benzene ligand [75]. A few years later, Williams et al. observed that this complex is highly luminescent at room temperature in diluted deoxygenated solution of dichloromethane (Φ_lum_ = 0.60 and τ = 7.2 μs) [76]. The discovery of its amazing luminescent properties, in which the rigidity of the terdentate ligand and the presence of a cyclometalating carbon have a positive effect [77], has been a springboard for the design of many platinum(II) complexes with variously substituted 1,3-bis(pyridin-2-yl)benzene ligands.

An interesting aspect of these Pt(bpyb)Cl derivatives is that the absorption and emission wavelengths depend on the presence of electron-donating and electron-withdrawing groups on the cyclometalating benzene and on the pyridine rings. Thus, time-dependent density functional theory calculations predict that the introduction of electron-withdrawing groups in the benzene ring and/or electron-donating groups in the pyridine rings gives a blue-shift in the emission, whereas the addition of electron-donating groups in the benzene ring and/or electron-withdrawing groups in the pyridines leads to a red-shift [78]. This behavior has been confirmed experimentally, offering a useful way to tune the color of the emission [58,77,79,80].

Recently, we found that the introduction of a variously substituted phenyl group on the position 4 of the pyridine rings of the *N^C^N* 1,3-bis(pyridin-2-yl)benzene ligand opens the door to a new family of luminescent platinum(II) complexes with quantum yields approaching unity [39,81,82,83], which has already found application in the fabrication of efficient OLEDs [39,81]. This observation prompted us to study the influence of the introduction of a different polarizable aromatic substituent, namely a 4-hexyl-2-thienyl group, on the position 4 of the pyridine rings.

In addition, it was reported that the introduction of a bulky mesityl group at position 5 of the cycloplatinating benzene ring of the 1,3-bis(pyridin-2-yl)-benzene ligand allows the tendency to form bi-molecular states to decrease due to steric hindrance. which inhibits the face-to-face approach of two platinum complexes, decreases Pt–Pt interactions and/or π–π stacking, and reduces self-quenching at elevated concentrations [84]. Such a steric effect is an interesting tool for the fabrication of OLEDs, in which local concentrations can be elevated, because self-quenching is an energy sink that reduces device efficiencies [84]. Remarkably, it was shown that substitution of the mesityl group at position 5 of the cycloplatinating benzene ring with a 2-thienyl group leads to a strong red-shift in the emission in line with the influence of the aromatic substituents on the energy of the HOMO [84].

**Chart 1:**
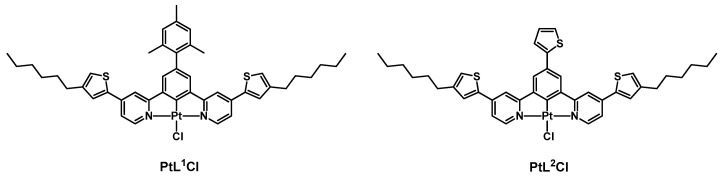
Chemical structures of the investigated Pt(II) complexes.

These reports, and the fact that the availability of complexes with tunable emission over a wide color range offers considerable scope in the development of OLEDs encouraged us to prepare and well-characterize Pt(1,3-bis(4-(4-hexyl-2-thienyl)-pyridin-2-yl)-5-mesitylbenzene)Cl and Pt(1,3-bis(4-(4-hexyl-2-thienyl)-pyridin-2-yl)-5-(2-thienyl)benzene)Cl (Chart 1). Both are characterized by excellent quantum yields.

## 2. Results and Discussion

### 2.1. Synthesis of the Platinum(II) Complexes

**Scheme 1:**
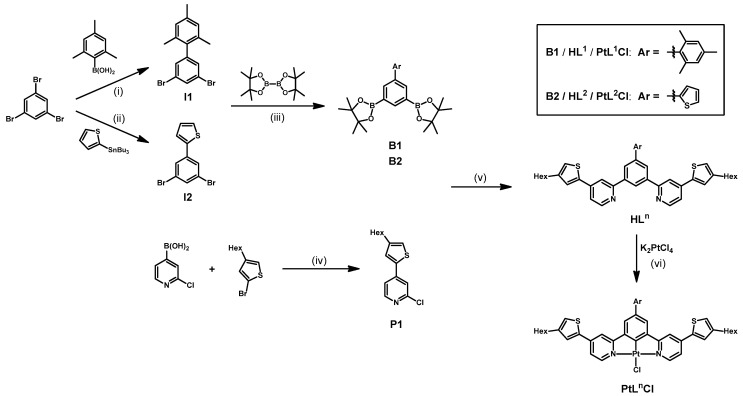
Synthesis of complexes **PtL^1^Cl** and **PtL^2^Cl**. (i): Pd(PPh_3_)_4_, Ba(OH)_2_·8H_2_O, 1,2-dimethoxyethane/H_2_O, Ar, 80 °C, 20 h; (ii): Pd(PPh_3_)_4_, LiCl, toluene, Ar, reflux, 24 h; (iii): Pd(dppf)Cl_2_, AcOK, toluene, Ar, reflux, 24 h; (iv): Pd(PPh_3_)_4_, Na_2_CO_3_, dioxane/H_2_O, Ar, 100 °C, 48 h; (v): Pd(PPh_3_)_4_, Na_2_CO_3_, 1,2-dimethoxyethane/H_2_O, Ar, 100 °C, 20 h; (vi): AcOH, Ar, reflux, 48 h.

The new pro-ligands, 1,3-bis(4-(4-hexyl-2-thienyl)-pyridin-2-yl)-5-mesitylbenzene (HL^1^) and 1,3-bis(4-(4-hexyl-2-thienyl)-pyridin-2-yl)-5-(2-thienyl)benzene (HL^2^), were synthesized by Suzuki–Miyaura cross-coupling the corresponding boronic acid pinacol ester [39] with 2-chloro-4-(4-hexyl-2-thienyl)-pyridine (Scheme 1). Reaction in glacial acetic acid of these pro-ligands with K_2_PtCl_4_ led to the desired complexes in 61–68% yield. They were fully characterized by NMR (^1^H and ^13^C) and elemental analysis. Full details of the synthesis and characterization are provided in the Section 3 and in the Supporting Information.

### 2.2. Photophysical Properties

**Figure 1:**
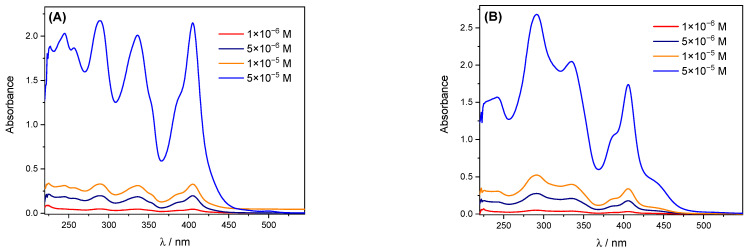
Absorption spectra of **PtL^1^Cl** (**A**) and **PtL^2^Cl** (**B**) in CH_2_Cl_2_ at different concentrations at 298 K.

The electronic absorption spectra of complexes **PtL^1^Cl** and **PtL^2^Cl** in CH_2_Cl_2_ at different concentrations (1 · 10^−6^–5 · 10^−5^ M) are presented in Figure 1. Both complexes are characterized by bands of high intensity below 330 nm, attributed to intraligand ^1^ π–π * transitions of the *N^C^N* cyclometalating ligand, analogous to related platinum(II) complexes [84]. The absorption bands at 330–470 nm correspond to transitions of mixed charge-transfer/ligand-centered character, as reported in the literature [84,85]. Accordingly, they exhibit a pronounced hypsochromic effect typical of electronic transitions with an appreciable degree of charge-transfer character; the blue-shift in the peaks with increasing solvent polarity (from toluene to dichloromethane) is in agreement with a higher dipole moment in the ground state than in the excited state [84] (see Figures S20 and S21).

**Figure 2:**
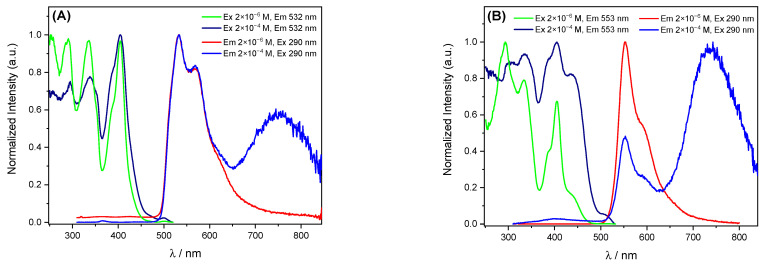
Normalized excitation and emission spectra of **PtL^1^Cl** (**A**) and **PtL^2^Cl** (**B**) at different concentrations in deaerated CH_2_Cl_2_ at 298 K.

The normalized excitation and emission spectra at room temperature of **PtL^1^Cl** and **PtL^2^Cl** in degassed dilute (2 · 10^−6^ M) and concentrated (2 · 10^−4^ M) dichloromethane solution are shown in Figure 2. After excitation at 290 nm in dilute solution, the emission spectrum of **PtL^1^Cl** shows a structured broad band characterized by a high-energy emission maximum at 534 nm, with shoulders at 568 and 617 nm, attributed to emission from the T_1_ state [82,84]. Complex **PtL^2^Cl** is characterized by a high-energy emission maximum at 554 nm, with a shoulder at 589 nm, red-shifted with respect to **PtL^1^Cl**, in agreement with the substitution of the mesityl group with the more-donating 2-thienyl moiety [84]. The same hypsochromic effect observed in the absorption spectra in toluene vs. dichloromethane is also observed in the excitation spectra (see Figures S24 and S25).

It is worth pointing out that the high-energy emission maximum of both complexes is red-shifted compared with the parent complexes with unsubstituted pyridine rings [84], which highlights the effect of introducing 4-hexyl-2-thienyl substituents. The effect is stronger in the presence of the mesityl group, although it is slightly less pronounced than that observed in the case of a triphenylamine substituent on the pyridine rings [81] (Table 1).

As expected from the typical comportment of *N^C^N* platinum complexes [30,79,80,81,82,83,84], an increase in the concentration of **[PtL^1^Cl]** and **[PtL^2^Cl]** up to 2 · 10^−4^ M leads to the appearance of a new, broad, structureless emission band centered at 752 and 738 nm, respectively, which becomes progressively more intense at the expense of the shorter wavelength bands. This lower energy band, ascribed to bi-molecular emissive excited states (excimers/aggregates) of the platinum (II) complexes generated through π–π ligand–ligand and Pt–Pt intermolecular interactions [28], is much more intense for **[PtL^2^Cl]** than for **[PtL^1^Cl]** (see Figure 2), highlighting that the presence of the bulky mesityl group in *para* of the cyclometalating benzene is much more efficient than the thienyl group in hampering the formation of dimeric species.

Both new complexes exhibit excellent absolute quantum yields, 0.96 and 0.99 for **PtL^1^Cl** and **PtL^2^Cl**, respectively, as measured with an integrating sphere (see Table 1). When the dichloromethane solution is aerated, the Φ_lum_ diminishes drastically to 0.02 and 0.06, due to oxygen quenching, in agreement with the behavior of related complexes (Table 1) [81,84]. This represents an interesting aspect for photodynamic therapy, because an effective generation of singlet oxygen can be expected for these complexes [71].

It is well-established that an augmentation of concentration typically causes a strong diminution of the luminescence quantum yield of *N^C^N* cyclometalated platinum(II) complexes due to the formation of excimers and aggregates [77]. In the case of **PtL^1^Cl** and **PtL^2^Cl**, although the quantum yield decreases upon increasing the concentration of the degassed dichloromethane solution, it remains very high (Φ_lum_ = 0.50 and 0.42 at 2 · 10^−4^ M, respectively), as previously observed for the complex related to **PtL^1^Cl** bearing a triphenylamine instead of a 4-hexyl-2-thienyl substituent (Φ_lum_ = 0.44 at 2 · 10^−4^ M) [81] and for other *N^C^N* cyclometalated platinum(II) complexes bearing a variously substituted phenyl group in position 4 of the pyridine rings [39,82,83]. This observation suggests that the introduction of aromatic groups on the pyridine rings is a route to maintain excellent quantum yields even in concentrated solution, an aspect of particular interest for the fabrication of OLEDs, in which local concentrations can be elevated.

Excited state decay measurements of **PtL^1^Cl** in degassed dilute dichloromethane solution were carried out, exciting at 405 nm at the emission wavelength of 532 nm, evidencing a luminescence lifetime, τ, of 24.1 μs (Table 1). A similar lifetime was obtained at the emission wavelength of 725 nm (ESI, Figure S20), in agreement with the tail of the emission spectra of the complex at low frequency in dilute solution (Figure 2). A much lower τ (6.1 μs) is obtained in concentrated solution (ESI, Figure S21), as expected, because an increase in the concentration leads to the formation of bi-molecular states with a diminution of both lifetime and quantum yield. Similarly, **PtL^2^Cl** is characterized by a longer luminescence lifetime in dilute (τ = 5.9 μs, Table 1) than in concentrated (τ = 1.4 μs) solution (ESI, Figures S22 and S23). It is worth pointing out that **PtL^1^Cl,** bearing a mesityl group in position 5 of the cyclometalating benzene, is characterized by a much longer lifetime than the related complex with non-substituted pyridine rings [84], revealing the positive effect of the introduction of 4-hexyl-2-thienyl substituents, which remains less pronounced than that observed in the case of triphenylamine substituents (Table 1). However, such an effect, though of interest for many applications like bioimaging [81], cannot be generalized because it is not observed when the mesityl group is substituted by a 2-thienyl one.

Moreover, the two new complexes were investigated in poly(methyl methacrylate)—PMMA—thin films with a loading of 1% *w*/*w*, registering the absorption and luminescence spectra, and measuring absolute quantum yields and lifetimes in air. In agreement with the relatively low amount of compound in the polymer, the UV–Vis absorption spectra present the same features of those of diluted solutions. Emission and excitation spectra are reported in Figures S32 and S33 and resemble the corresponding spectra in dichloromethane solution, as expected as a consequence of the low loading of the complex in the polymer, so that no aggregate species are clearly visible. The luminescence quantum yields of the films are 0.50 and 0.40, for **PtL^1^Cl** and **PtL^2^Cl**, respectively, similar to that (0.56) previously reported for a related Pt(II) complex with triphenylamino substituents on the pyridine rings [81]; these quantum yields are remarkable since the measurements are carried out in air. With respect to solutions, the lifetimes decrease in the PMMA films (16.4 vs. 24.1 μs for **PtL^1^Cl**, and 5.1 vs. 5.9 μs for **PtL^2^Cl**). In addition, kr values of 3.03 · 10^4^ s^−1^ (**PtL^1^Cl**) and 7.78 · 10^4^ s^−1^ (**PtL^2^Cl**) were found, which are interesting values for the preparation of OLED devices (see Table S3 for complete data). The calculated k_r_ are quite similar to those found for dilute dearated solutions, while k_nr_ are higher, due to the vibrational motions induced by the interaction with the PMMA matrix.

### 2.3. Theoretical Calculations

**Figure 3:**
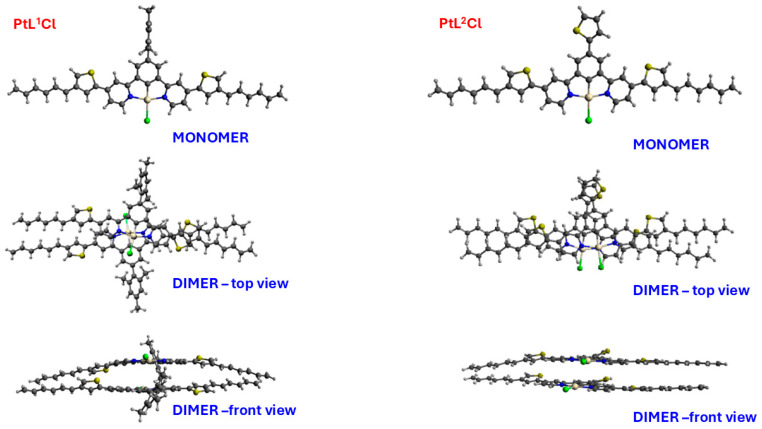
Optimized geometries of the monomers and dimers of **PtL^1^Cl** and **PtL^2^Cl** in their top and front views.

To give insight into the electronic and optical properties of the two investigated complexes, we employed computational modeling with methods based on the density functional theory (DFT) and its time-dependent version (TDDFT). We started by optimizing the molecular geometry of the two monomeric complexes and of their dimers by including dichloromethane solvation effects. The optimized geometries of the two investigated Pt complexes and their related dimers are reported in Figure 3. For the dimer of **PtL^1^Cl**, with the bulky mesityl group at position 5 of the cycloplatinating benzene ring, only one dimer molecular geometry characterized by the mesityl group on the opposite side has been optimized. For compound **PtL^2^Cl,** we explored the two possible reciprocal arrangements of the two complex units, finding that in dichloromethane solution, the most stable configuration by 1.7 kcal/mol is that one with the two 4-hexyl-2-thienyl groups on the same side. We consider the optimized monomers representative of the low-concentration solution and the dimers of the higher concentrations. We have seen for similar systems that the considered models (monomer and dimer), though simplified, are able to describe two different concentration conditions [39].

We therefore simulated the spectra of complexes **PtL^1^Cl** and **PtL^2^Cl**, both as monomers and dimers, by computing 60 TDDFT excitations for the monomers and 150 excitations for the dimers, thus, we are able to describe the 450–270 nm and 480–290 nm regions of the absorption spectra of the monomers and dimers, respectively. All the simulated absorption spectra are presented in Figure S36.

**Figure 4:**
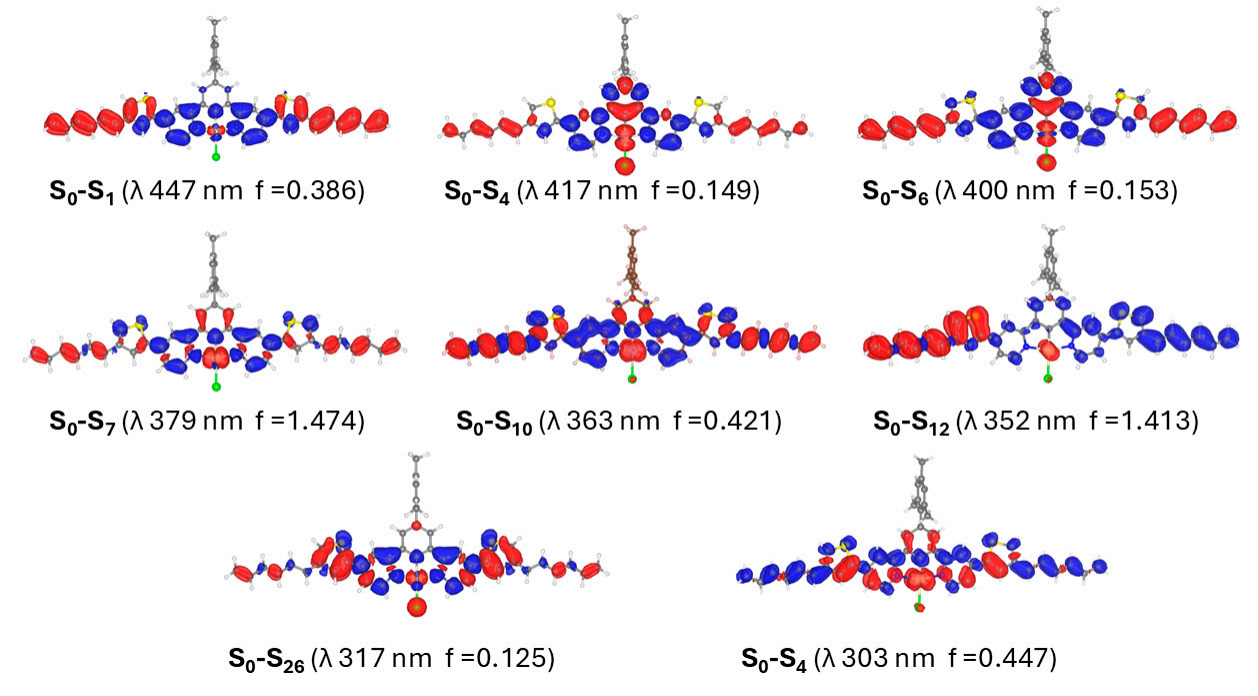
Electron density differences in the most relevant S_0_–S_n_ electronic transitions of the **PtL^1^Cl** monomer.

**Figure 5:**
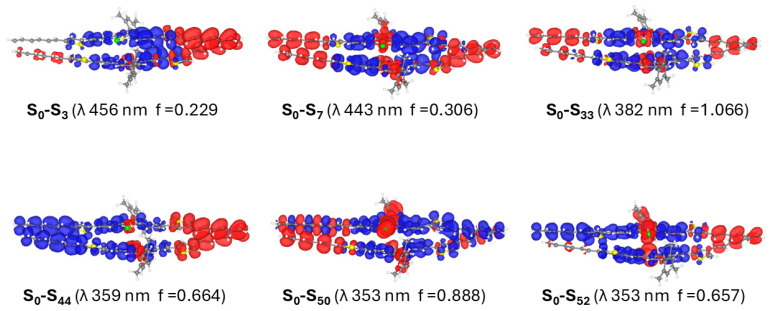
Electron density differences in the most relevant S_0_–S_n_ electronic transitions of the **PtL^1^Cl** dimer.

**Figure 6:**
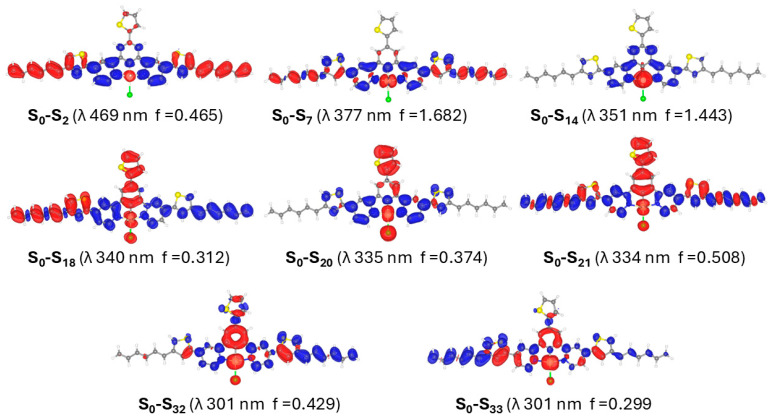
Electron density differences in the most relevant S_0_–S_n_ electronic transitions of the **PtL^2^Cl** monomer.

**Figure 7:**
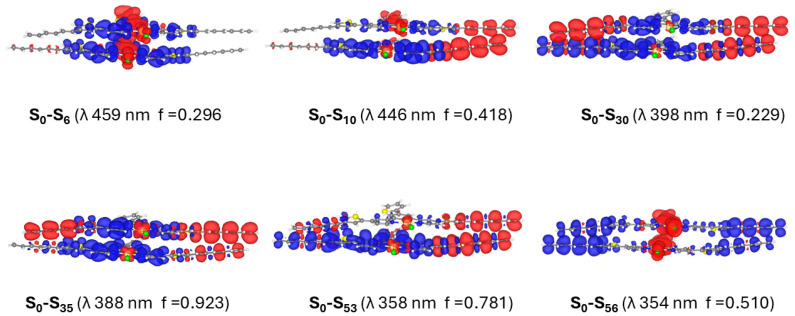
Electron density differences in the most relevant S_0_–S_n_ electronic transitions of the **PtL^2^Cl** dimer.

We observe that the spectra of the monomers of **PtL^1^Cl** and **PtL^2^Cl** are quite similar in the 400–320 nm region, while a few differences essentially due to a higher intensity of the transitions computed for the **PtL^2^Cl** monomer are evidenced in the 400–500 nm region and 320–290 nm region. The spectra of dimers show a few differences due to the higher intensity of the transitions of **PtL^1^Cl** dimer and for a larger number of transitions characterized by a comparable or lower intensity in the considered wavelength range. The reasonable agreement between the measured and simulated UV–Vis spectra prompted us to investigate the nature of the main transitions giving rise to the spectra in order to assign the spectral bands. To this end, we calculated the electron density differences between the most intense excited states and the ground state electron density for both monomers and dimers. This kind of analysis provides a valuable tool for assigning the character of spectral bands since it visualizes the directionality of the electron density from the initial state to the final excited state. In Figure 4, Figure 5, Figure 6 and Figure 7, the plots of the density difference between the excited states and the ground state are reported for both **PtL^1^Cl** and **PtL^2^Cl** in the monomer and dimer forms: in red, where the electronic density moves is reported, and in blue, where it arrives. We have considered the excited states with the highest oscillator strengths that mainly contribute to the main spectral features. It is interesting to notice that the main transition involves as starting states the Pt d orbitals mixed with π combinations on the substituted 2-pyridyl-benzene ligands, while the arriving states are delocalized on the antibonding π combinations of the Pt ligands. This observation brings us to assign the transitions generating the spectra as metal-to-ligand charge-transfer transitions (MLCT) and metal–ligand-to-ligand charge-transfer transitions (MLLCT).

## 3. Materials and Methods

All the reagents and the solvents were used as received from the supplier (Merck, Rahway, NJ, USA). The purifications were performed through column chromatography on silica gel (Merck Geduran 60, 0.063–0.200 mm).

The NMR characterizations were obtained recording on a Bruker AV III 300 MHz or AV III 400 MHz spectrometers (Billerica, MA, USA). Chemical shifts in ^1^H and ^13^C NMR spectra are reported in parts per million (ppm) and the coupling constants are measured in Hertz (Hz). The multiplicities of signals are listed as singlet (s), d (doublet), t (triplet), quartet (q), multiplet (m).

Elemental analyses were performed by the Department of Chemistry of the University of Milan.

Electronic absorption spectra in solution were obtained with a UV-3600i Plus UV-VIS-NIR spectrophotometer (Shimadzu Italia S.r.l., Milan, Italy). Luminescence measurements were carried out in CH_2_Cl_2_ solution after the Freeze–Pump–Thaw (FPT) procedure, necessary to remove dissolved oxygen. Absolute photoluminescence quantum yield, Φ, was measured using a C11347 Quantaurus Hamamatsu Photonics K.K spectrometer (Hamamatsu, Japan, See ESI)_._ The details about the synthesis of intermediated **I1** and **I2**, of the thienyl-pyridine **P1** and of boronic esters **B1** and **B2** are reported in the ESI, together with the NMR spectra of all compounds.

### 3.1. Synthesis of PtL^1^Cl

#### 3.1.1. Synthesis of Ligand HL^1^

Boronic ester **B1** (32 mg, 0.069 mmol), pyridine **P1** (58 mg, 0.207 mmol), Na_2_CO_3_ (54 mg, 0.514 mmol) and Pd(PPh_3_)_4_ (6 mg, 0.005 mmol) were added to 1,2-dimethoxyethane (1.0 mL) and water (1.0 mL) in a Schlenk tube and the mixture was stirred at reflux under Ar atmosphere. After 48 h, the reaction was cooled to rt, AcOEt and water were added, and the phases were separated. The organic phase was washed with water, and the aqueous phase was extracted with AcOEt; the organic phases were dried over Na_2_SO_4_ and evaporated at reduced pressure. The reaction mixture was purified by flash chromatography on silica gel (eluent: hexane/AcOEt 9:1), obtaining 21 mg of product (yield 45%).

^1^H-NMR (400 MHz, CD_2_Cl_2_) δ (ppm): 8.88 (1H, bs), 8.71 (2H, d, J = 5.2 Hz), 8.11 (2H, s), 7.95 (2H, d, J = 1.3 Hz), 7.57 (2H, s), 7.52 (2H, dd, J = 1.3 Hz, J = 5.2 Hz), 7.11 (2H, s), 7.04 (2H, s), 2.69 (4H, t, J = 7.6 Hz), 2.39 (3H, s), 2.15 (6H, s), 1.75–1.67 (4H, m), 1.43–1.32 (12H, m), 0.93 (6H, t, J = 6.8 Hz).

^13^C-NMR (100 MHz, CD_2_Cl_2_) δ (ppm): 149.52, 128.86, 128.09, 127.46, 124.24, 122.46, 118.56, 116.83, 31.63, 30.44, 30.40, 28.93, 22.59, 20.74, 20.64, 13.81.

#### 3.1.2. Synthesis of Complex PtL^1^Cl

Ligand **HL^1^** (21 mg, 0.031 mmol) and K_2_PtCl_4_ (15 mg, 0.036 mmol) were added to glacial AcOH (1.0 mL) in a Schlenk tube and the mixture was stirred at reflux under Ar atmosphere. After 48 h, the reaction was cooled to rt and water was added to precipitate more product. The orange precipitate was filtered on a Buchner funnel, washed with H_2_O, MeOH and Et_2_O, and dried, obtaining 17 mg of product (yield 61%) which was not further purified.

^1^H-NMR (400 MHz, CD_2_Cl_2_) δ (ppm): 9.20 (2H, d, J = 6.1 Hz, J(^195^Pt) = 40 Hz), 7.86 (2H, d, J = 2.0 Hz), 7.55 (2H, d, J = 1.1 Hz), 7.49 (2H, dd, J = 2.0 Hz, J = 6.1 Hz), 7.41 (2H, s), 7.19 (2H, d, J = 1.1 Hz), 7.04 (2H, s), 2.69 (4H, t, J = 7.6 Hz), 2.39 (3H, s), 2.16 (6H, s), 1.74–1.64 (4H, m), 1.42–1.32 (12H, m), 0.93 (6H, t, J = 6.9 Hz).

^13^C-NMR (100 MHz, CD_2_Cl_2_) δ (ppm): 151.73, 145.55, 139.24, 136.05, 128.68, 128.05, 125.26, 124.32, 122.59, 118.59, 115.01, 31.61, 30.37, 28.89, 22.57, 20.66, 13.81.

### 3.2. Synthesis of PtL^2^Cl

#### 3.2.1. Synthesis of Ligand H^2^L

Boronic ester **B2** (43 mg, 0.104 mmol), pyridine **P1** (87 mg, 0.312 mmol), Na_2_CO_3_ (81 mg, 0.768 mmol) and Pd(PPh_3_)_4_ (8 mg, 0.007 mmol) were added to 1,2-dimethoxyethane (1.5 mL) and water (1.5 mL) in a Schlenk tube and the mixture was stirred at reflux under Ar atmosphere. After 48 h, the reaction was cooled to rt, AcOEt and water were added and the phases were separated. The organic phase was washed with water, and the aqueous phase was extracted with AcOEt; the organic phases were dried over Na_2_SO_4_ and evaporated at reduced pressure. The reaction mixture was purified by flash chromatography on silica gel (eluent: hexane/AcOEt 8:2); since the obtained product was not sufficiently pure, a preparative TLC was then performed eluent: hexane/AcOEt 9:1), obtaining 10 mg of product (yield 15%).

^1^H-NMR (400 MHz, CD_2_Cl_2_) δ (ppm): 8.74 (2H, d, J = 5.2 Hz), 8.86 (1H, t, J = 1.5 Hz), 8.42 (2H, d, J = 1.5 Hz), 8.12 (2H, d, J = 0.9 Hz), 7.63 (1H, d, J = 3.6 Hz), 7.58 (2H, d, J = 1,1 Hz), 7.54 (2H, dd, J = 0.9 Hz, J = 5.2 Hz), 7.43 (1H, d, J = 5.1 Hz), 7.21 (1H, dd, J = 3.6 Hz, J = 5.1 Hz), 7.13 (2H, s), 2.71 (4H, t, J = 7.6 Hz), 1.77–1.66 (4H, m), 1.46–1.33 (12H, m), 0.94 (6H, t, J = 7.1 Hz).

^13^C-NMR (100 MHz, CD_2_Cl_2_) δ (ppm): 156.99, 149.77, 145.14, 143.79, 143.01, 140.55, 140.11, 135.44, 128.19, 127.34, 125.38, 125.27, 124.80, 124.07, 122.41, 118.73, 116.86, 31.65, 30.43, 30.42, 28.96, 22.61, 13.84.

#### 3.2.2. Synthesis of Complex PtL^2^Cl

Ligand **HL^2^** (10 mg, 0.015 mmol) and K_2_PtCl_4_ (8 mg, 0.019 mmol) were added to glacial AcOH (0.5 mL) in a Schlenk tube and the mixture was stirred at reflux under Ar atmosphere. After 48 h, the reaction was cooled to rt and water was added to precipitate more product. The orange precipitate was filtered on a Buchner funnel, washed with H_2_O, MeOH and Et_2_O, and dried, obtaining 9 mg of product (yield 68%) which was not further purified.

^1^H-NMR (400 MHz, CD_2_Cl_2_) δ (ppm): 9.15 (2H, d, J = 6.1 Hz, J(^195^Pt) = 39 Hz), 7.89 (2H, s), 7.81 (2H, s), 7.59 (2H, s), 7.54 (1H, d, J = 3.5 Hz), 7.47–7.39 (3H, m), 7.23–7.18 (3H, m), 2.71 (4H, t, J = 7.7 Hz), 1.76–1.67 (4H, m), 1.47–1.34 (12H, m), 0.95 (6H, t, J = 6.7 Hz).

### 3.3. Preparation of PMMA Films

Thin films containing 1 wt% of complexes **PtL^1^Cl** and **PtL^2^Cl** in poly-methylmethacrylate (PMMA, Mw ≈ 15,000 g/mol) on quartz plate (thickness 1 mm) were obtained by spin-coating (Cookson Electronic Company P-6708D, Phoenix, AZ, USA). The parameters of spinning (RPM-revolutions per minute) were RPM 1: 800; Ramp 1: 1 s, Time 1: 5 s; RPM 2: 2000; Ramp 2: 1 s, Time 2: 120 s; RPM 3: 4000; Ramp 3: 2 s, Time 30 s. The solutions for the 1 wt% thin film were prepared with 1.33 mg of complex and 133 mg of PMMA in 1 mL of dichloromethane.

### 3.4. Theoretical Calculations

The geometry optimizations of monomers and dimers of complexes **PtL^1^Cl** and **PtL^2^Cl** were performed with the Gaussian09 program package (G09) [86] by using the B3LYP exchange–correlation functional [87] integrated with the D3-BJ model [88] to include the dispersion effects in the geometry optimizations. The dichloromethane solvation effects were included through the conductor-like polarizable continuum model as implemented in G09 [89,90,91,92]. All the atoms except Pt were described by 6–31G** basis set [93,94,95], while Pt was described with the LANL2DZ basis set, along with the corresponding pseudopotentials [96]. A total of 60 and 150 TDDFT excitations were computed for the **PtL^1^Cl** and **PtL^2^Cl** monomers and dimers, respectively, by including dichloromethane solvation effects.

## 4. Conclusions

In conclusion, two new complexes, namely Pt(1,3-bis(4-(4-hexyl-2-thienyl)-pyridin-2-yl)-5-mesitylbenzene)Cl and Pt(1,3-bis(4-(4-hexyl-2-thienyl)-pyridin-2-yl)-5-(2-thienyl)benzene)Cl were easily synthesized and well-characterized. In degassed diluted dichloromethane solution, both exhibit luminescence quantum yield approaching unity (Φ_lum_ = 0.96–0.99) in the green region of the visible spectrum (534–554 nm) with lifetimes values of 24.1 and 5.9 µs, respectively. Interestingly, this work shows that the introduction of a 2-thienyl group on the pyridine rings is a route to maintain very good quantum yields (Φ_lum_ = 0.42–0.50) even in concentrated solution, a feature of particular interest for the preparation of OLEDs. Also, it confirms that a bulky mesityl group in position 5 of the cyclometalating benzene hampers the neighbouring of monomeric complexes better than a 2-thienyl group, as evidenced by the lower intensity of the low energy band characteristic of excimers/aggregates in concentrated solutions. These observations represent guidelines for the design of *N^C^N* platinum complexes, which are of importance for the development of luminescent materials [97,98]. As a next step, it would be of interest to use the novel complexes bearing a long alkyl chain, as prepared in the present work, as solution-processable emitters for the fabrication of solution-processed OLEDs.

## Figures and Tables

**Table 1:** Key photophysical parameters of **PtL^1^Cl** and **PtL^2^Cl** in comparison with that of related complexes [81,84], at 298 K in dilute degassed CH_2_Cl_2_ solution.

	λ_max,em/_nm Monomer [Excimer/Aggregate] ^a^	Φ_lum_ Degassed [Aerated]	τ/µs
** 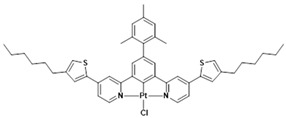 **	534 ^b^ [752]	0.96 ^b^ [0.02]	24.1
** 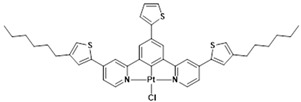 **	554 ^b^ [738]	0.99 ^b^ [0.06]	5.9
** 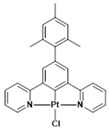 **	501 [690–700]	0.62 ^c^ [0.045]	7.9
** 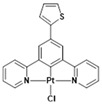 **	548 [690–700]	0.54 ^c^ [0.015]	20.5
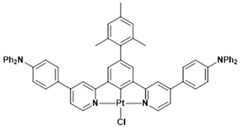	548 [725]	0.89 ^b,d^ [0.01]	50.0

^a^ Excimer/aggregate in concentrated solution. ^b^ Absolute Φ_lum_ measured with an integrating sphere. ^c^ Luminescence quantum yields were determined by the method of continuous dilution, using quinine sulfate in 1 M H_2_SO_4_ (Φ_lum_ = 0.546) as the standard; the estimated uncertainty is 20% or better [84]. ^d^ From reference [81].

## Data Availability

The original contributions presented in this study are included in the article/Supplementary Material. Further inquiries can be directed to the corresponding authors.

## Supplementary Materials

The following supporting information can be downloaded at https://www.mdpi.com/article/10.3390/molecules30224410/s1, the full characterization of the two complexes (Synthetic procedures, ^1^H and ^13^C NMR spectra, photophysical characterization in solution and in thin films, calculated absorption spectra) have been included as part of the Supplementary Information. Reference [99] is cited in the Supplementary Materials.
