# Supplementary material for: Introduction of a 4-Hexyl-2-thienyl Substituent on Pyridine Rings as a Route for Brightly Luminescent 1,3-Di-(2-pyridyl)benzene Platinum(II) Complexes
Source: Molecules. 2025 Nov 14;30(22):4410. doi: 10.3390/molecules30224410 (PMC12655828; doi:10.3390/molecules30224410)
Supplement: Supplementary file 1 [file molecules-30-04410-s001.zip › molecules-3930327-supplementary.pdf]

Introduction of a 4-hexyl-2-thienyl substituent on pyridine rings as a route for brightly luminescent 1,3-di-(2-pyridyl)benzene platinum(II) complexes

Alessia Colombo,[1] Claudia Dragonetti,[1] Francesco Fagnani,[1,*] Dominique Roberto,[1,*] Simona Fantacci,[2] Daniele Marinotto[3]

[1]Dipartimento di Chimica, Università degli Studi di Milano and UdR-INSTM di Milano, Via C. Golgi 19, I-20133 Milan, Italy.

[2]Istituto di Scienze e Tecnologie Chimiche ‘‘Giulio Natta’’ SCITEC, Consiglio Nazionale delle Ricerche (CNR), Computational Laboratory for Hybrid/Organic Photovoltaics (CLHYO), via Elce di Sotto 8, 06213, Perugia, Italy.

[2]Istituto di Scienze e Tecnologie Chimiche (SCITEC) "Giulio Natta", Consiglio Nazionale delle Ricerche (CNR), via C. Golgi 19, I-20133 Milan, Italy.

1. Synthesis of the complexes

General comments

All the reagents and the solvents were used as received from the supplier. The purifications were performed through column chromatography on silica gel (Merck Geduran 60, 0.063–0.200 mm). The NMR characterizations were obtained recording on a Bruker AV III 300 MHz or AV III 400 MHz spectrometers. Chemical shifts of 1H and 13C NMR spectra are reported in parts per million (ppm) and the coupling constants are measured in Hertz (Hz). The multiplicities of signals are listed as singlet (s), d (doublet), t (triplet), quartet (q), multiplet (m).

Synthetic procedures

Synthesis of intermediate **I1**. 1,3,5-tribromobenzene (962 mg, 3.56 mmol), mesitylboronic acid (512 mg, 3.125 mmol), $Ba(OH)_2 \cdot 8H_2O$ (1445 mg, 4.575 mmol) and $Pd(PPh_3)_4$ (105 mg, 0.090 mmol) were added to 1,2-dimethoxyethane (6.0 mL) and water (1.0 mL) in a Schlenk tube and the mixture was stirred at 80 °C under Ar atmosphere. After 20 h the reaction was cooled to rt, toluene and water were added and the phases were separated. The organic phase was washed with brine (2x) and water (2x), dried over Na_2SO_4 and evaporated at reduced pressure. The reaction mixture was purified by flash chromatography on silica gel (eluent: hexane), obtaining 835 mg of product (yield 77%).
^{1}H-NMR (300 MHz, $CDCl_3$) δ (ppm): 7.66 (1H, t, J = 1.6 Hz), 7.26 (2H, d, J = 1.6 Hz), 6.94 (2H, s), 2.34 (3H, s), 2.02 (6H, s).

Synthesis of boronic ester **B1**. Intermediate **I1** (303 mg, 0.860 mmol), bis(pinacolato)diboron (477 mg, 1.878 mmol), AcOK (419 mg, 4.269 mmol) and $Pd(dppf)Cl_2$ (44 mg, 0.060 mmol) were added to dry toluene (3.0 mL) in a Schlenk tube and the mixture was stirred at reflux under Ar atmosphere. After 24 h the reaction was cooled to rt, AcOEt and water were added and the phases were separated. The organic phase was washed with water (2x) and the aqueous phase was extracted with AcOEt. The organic phases were dried over Na_2SO_4 and evaporated at reduced pressure. The reaction mixture was purified by flash chromatography on silica gel (eluent: hexane/AcOEt 9:1), obtaining 217 mg of product (yield 54%).
^{1}H-NMR (400 MHz, $CDCl_3$) δ (ppm): 8.25 (1H, t, J = 1.0 Hz), 7.70 (2H, d, J = 1.0 Hz), 6.90 (2H, s), 2.32 (3H, s), 1.99 (6H, s), 1.36 (24H, s).

Synthesis of intermediate **I2**. 1,3,5-tribromobenzene (453 mg, 1.439 mmol), 2-(tributyl)stannyl-thiophene (587 mg, 502 µL, 1.572 mmol), LiCl (304 mg, 7.162 mmol) and $PdCl_2(PPh_3)_2$ (49 mg,

0.070 mmol) were added to dry toluene (2.0 mL) in a Schlenk tube and the mixture was stirred at reflux under Ar atmosphere. After 24 h the solution was cooled to rt, the toluene was evaporated at reduced pressure, DCM and water were added and the phases were separated. The organic phase was dried over Na_2SO_4 and evaporated. The reaction mixture was purified by flash chromatography on silica gel (eluent: hexane/AcOEt from 8:2 to 6:4), obtaining 224 mg of product (yield 48%).
^{1}H-NMR (400 MHz, $CDCl_3$) δ (ppm): 7.69 (2H, s), 7.58 (1H, s), 7.37 (1H, d, J = 5.0 Hz), 7.33 (1H, d, J = 3.4 Hz), 7.11 (1H, dd, J = 3.4 Hz, J = 5.0 Hz).

Synthesis of boronic ester **B2**. Intermediate **I2** (150 mg, 0.472 mmol), bis(pinacolato)diboron (264 mg, 1.040 mmol), AcOK (232 mg, 2.364 mmol) and Pd(dppf)Cl_2 (24 mg, 0.033 mmol) were added to dry toluene (1.6 mL) in a Schlenk tube and the mixture was stirred at reflux under Ar atmosphere. After 24 h the reaction was cooled to rt, AcOEt and water were added and the phases were separated. The organic phase was washed with water (2x) and the aqueous phase was extracted with AcOEt. The organic phases were dried over Na_2SO_4 and evaporated at reduced pressure. The reaction mixture was purified by flash chromatography on silica gel (eluent: hexane/AcOEt 9:1), obtaining 86 mg of product (yield 44%).
^{1}H-NMR (400 MHz, $CDCl_3$) δ (ppm): 8.21 (1H, t, J = 1.0 Hz), 8.15 (2H, d, J = 1.0 Hz), 7.42 (1H, d, J = 3.6 Hz), 7.29 (1H, d, under residual $CHCl_3$), 7.09 (1H, dd, J = 3.6 Hz, J = 5.0 Hz).

Synthesis of pyridine **P1**. 2-bromo-4-hexylthiophene (589 mg, 482 μL, 2.382 mmol), 2-chloro-pyridine-4-boronic acid (250 mg, 1.589 mmol), Na_2CO_3 (508 mg, 4.793 mmol) and $Pd(PPh_3)_4$ (83 mg, 0.718 mmol) were added to 1,4-dioxane (9.0 mL) and water (3.0 mL) in a Schlenk tube and the mixture was stirred at reflux under Ar atmosphere. After 48 h water and AcOEt were added and the phases were separated. The organic phase was washed with water (3x), then dried over Na_2SO_4 and evaporated. The reaction mixture was purified by flash chromatography on silica gel (eluent: hexane/AcOEt from 9:1 to 8:2), obtaining 265 mg of product (yield 60%).
^{1}H-NMR (400 MHz, $CDCl_3$) δ (ppm): 8.35 (1H, d, J = 5.3 Hz), 7.51 (1H, d, J = 1.0 Hz), 7.41-7.36 (2H, m), 7.07 (1H, s), 2.65 (2H, t, J = 7.8 Hz), 1.71-1.61 (2H, m), 1.42-1.28 (6H, m), 0.92 (3H, t, J = 6.8 Hz).

2. NMR characterization

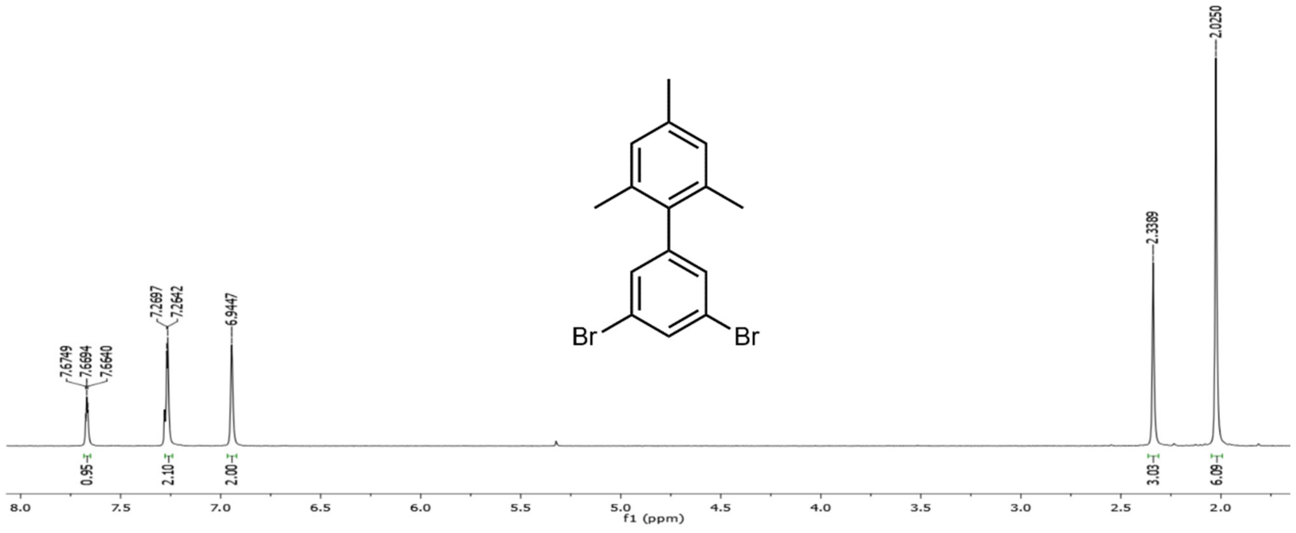

Figure S1. ^{1}H-NMR spectrum of **I1**.

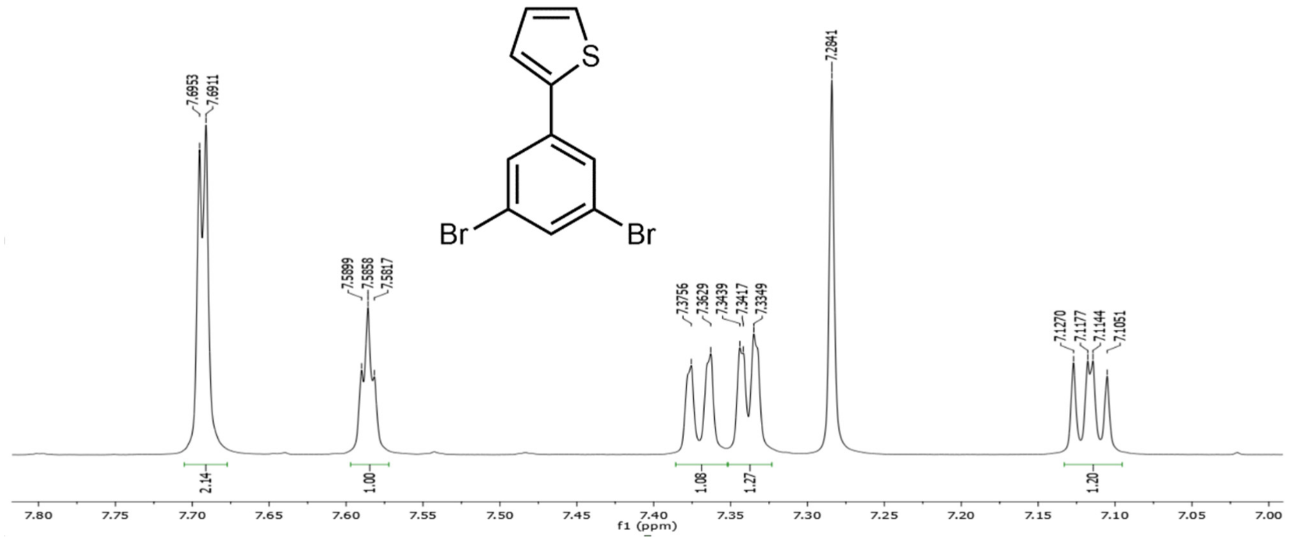

Figure S2. ^{1}H-NMR spectrum of **I2**.

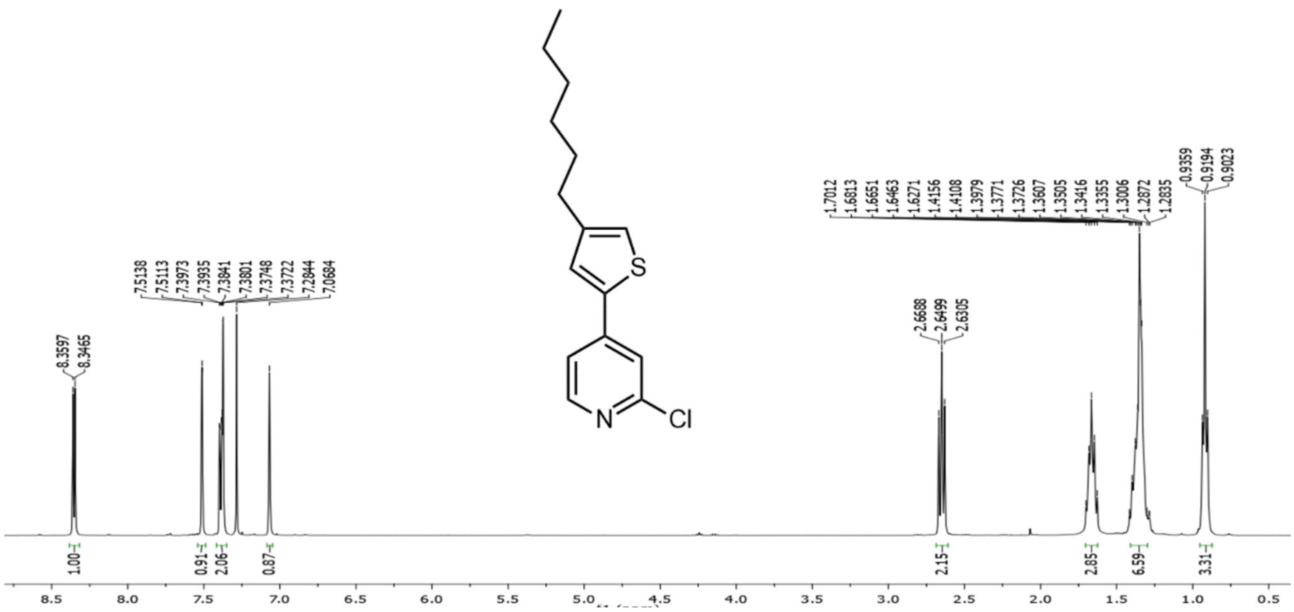

Figure S3. ^{1}H-NMR spectrum of **P1**.

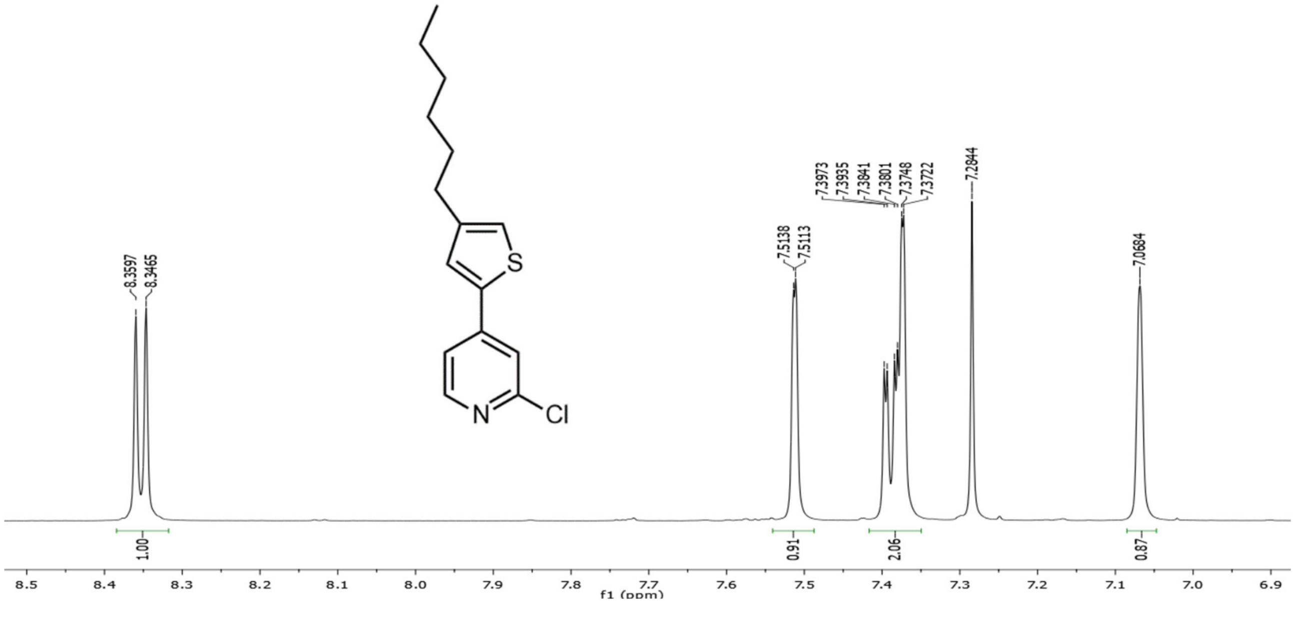

Figure S4. ^{1}H-NMR spectrum of **P1**, aromatic region.

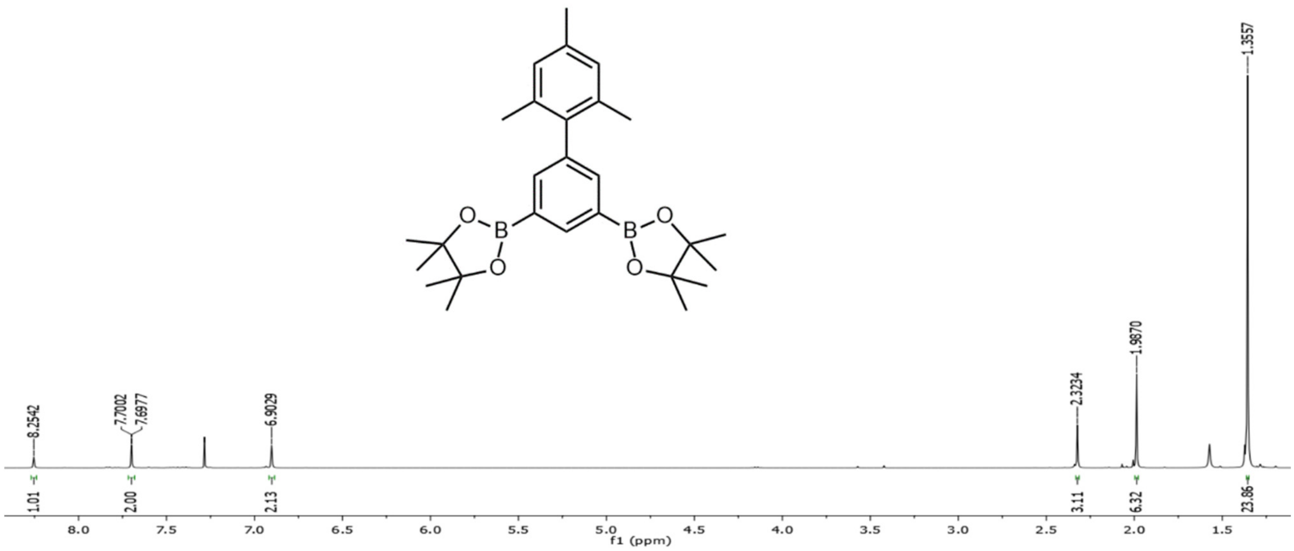

Figure S5. ^{1}H-NMR spectrum of **B1**.

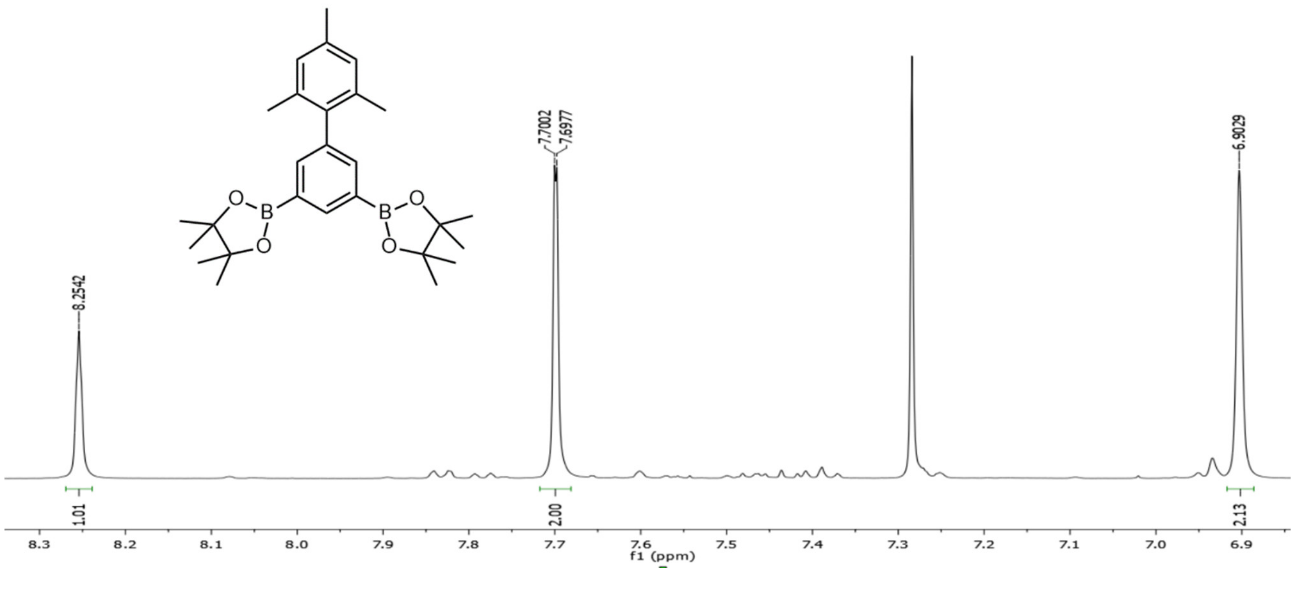

Figure S6. ^{1}H-NMR spectrum of **B1**, aromatic region.

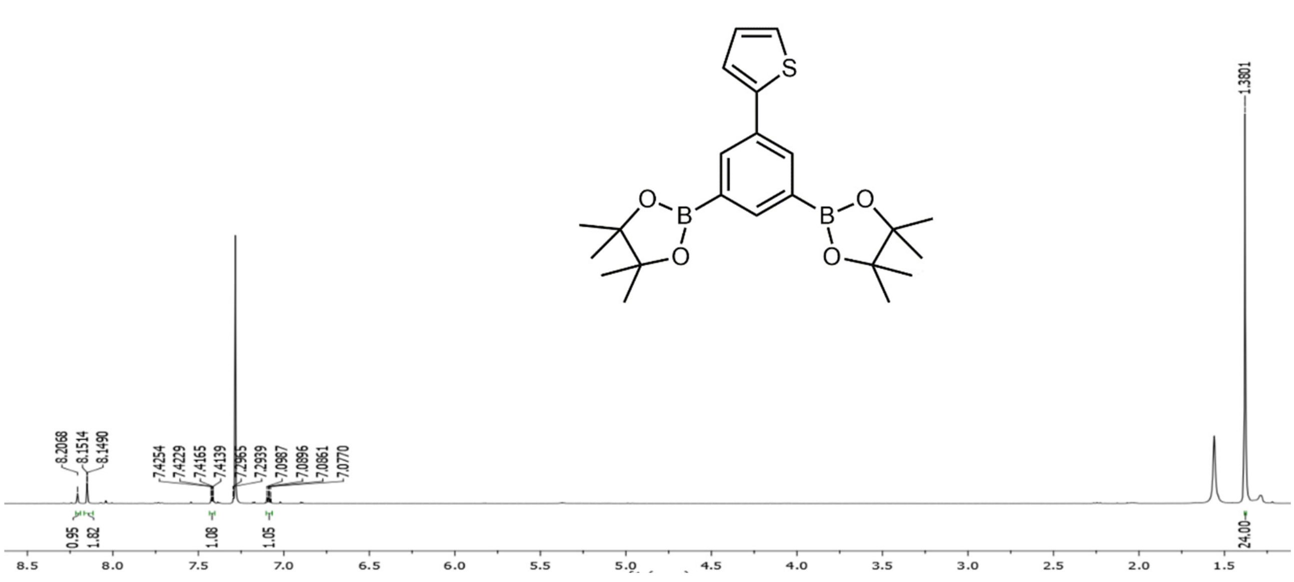

Figure S7. ^{1}H-NMR spectrum of **B2**.

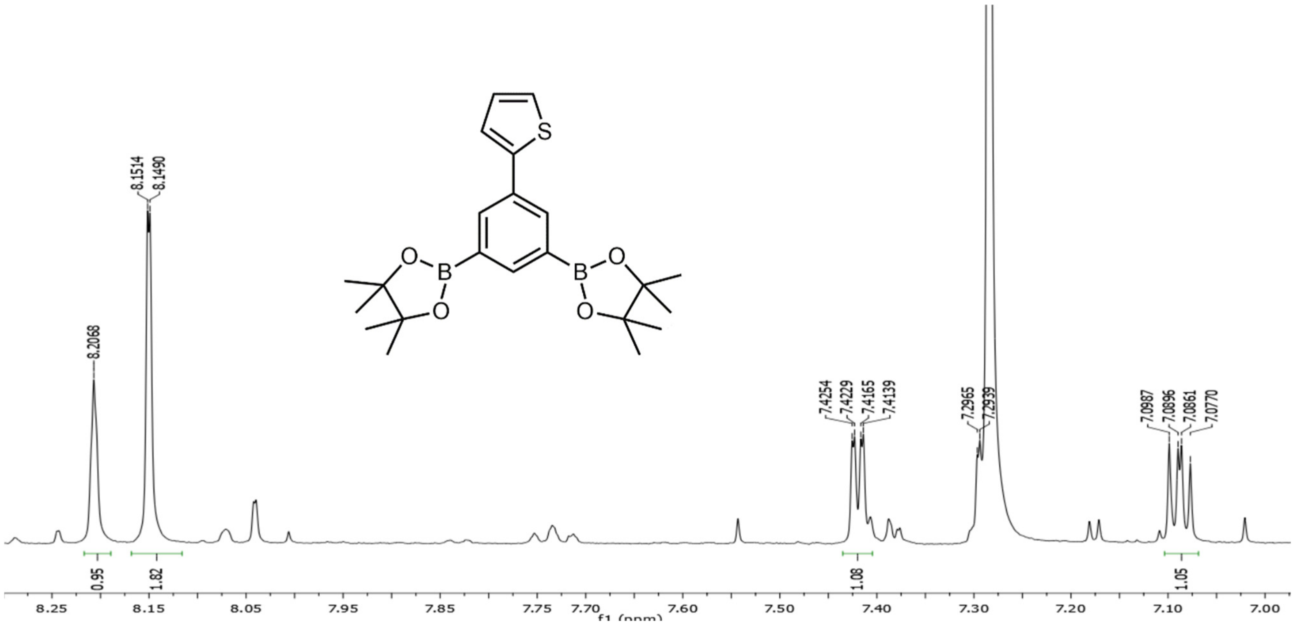

Figure S8. ^{1}H-NMR spectrum of **B2**, aromatic region.

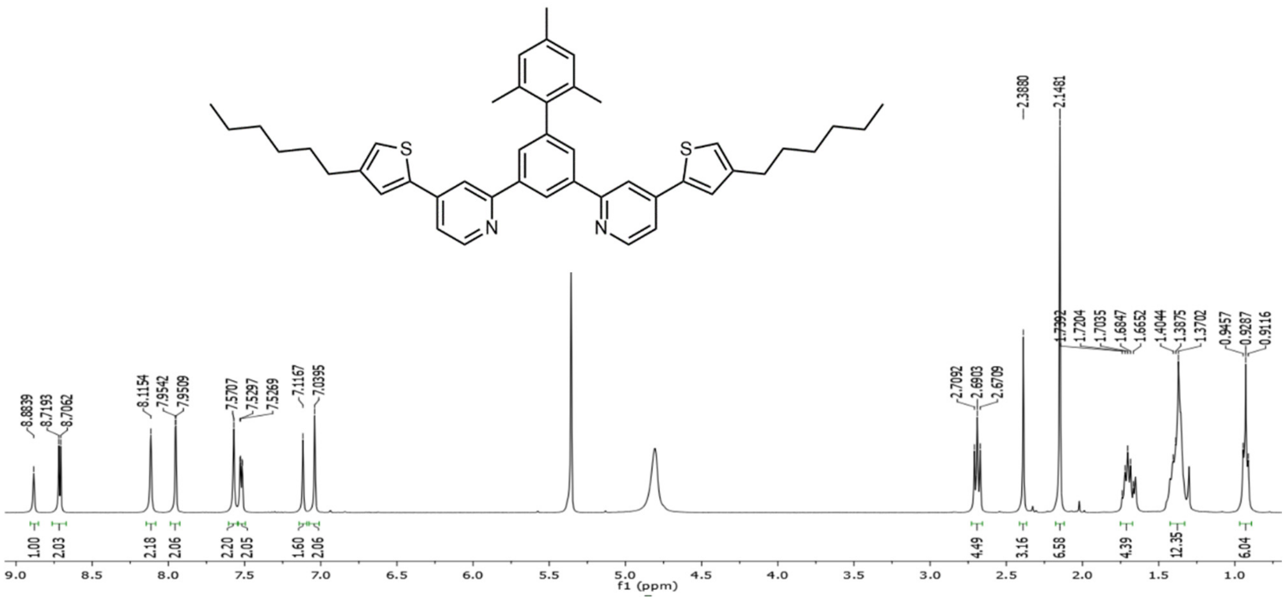

Figure S9. ^{1}H-NMR spectrum of ligand **HL^{1}**.

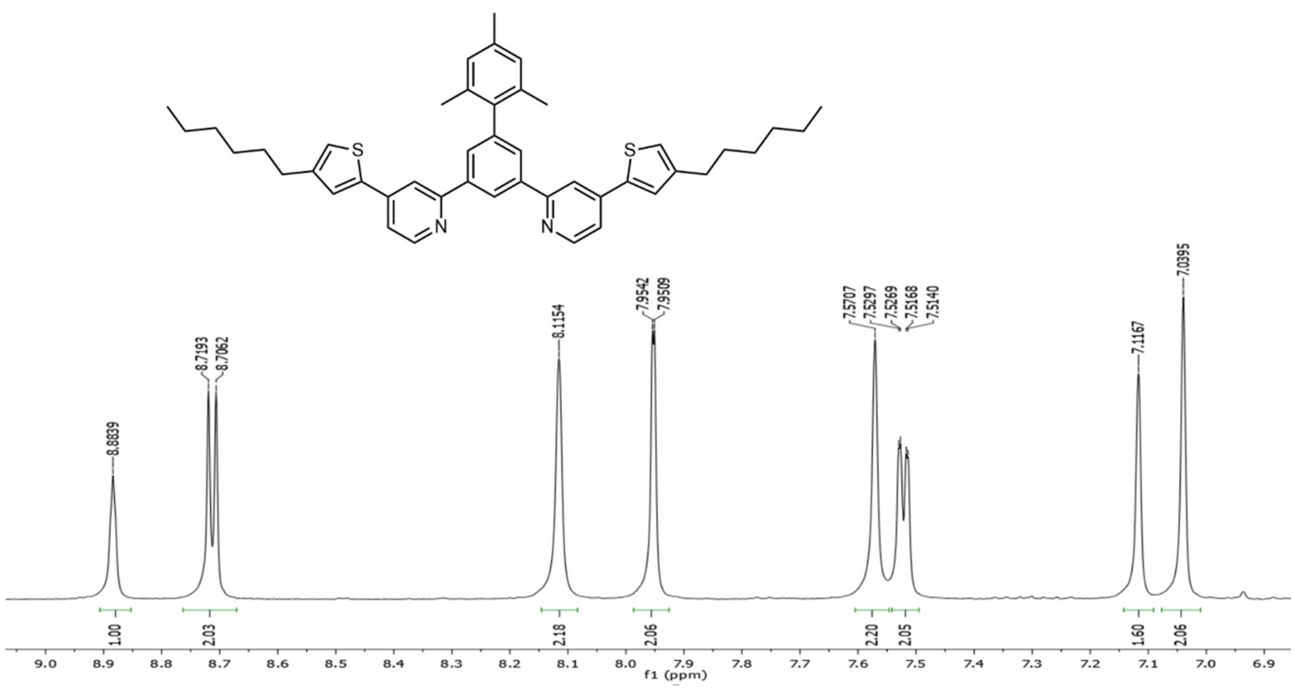

Figure S10. ^{1}H-NMR spectrum of ligand **HL^{1}**, aromatic region.

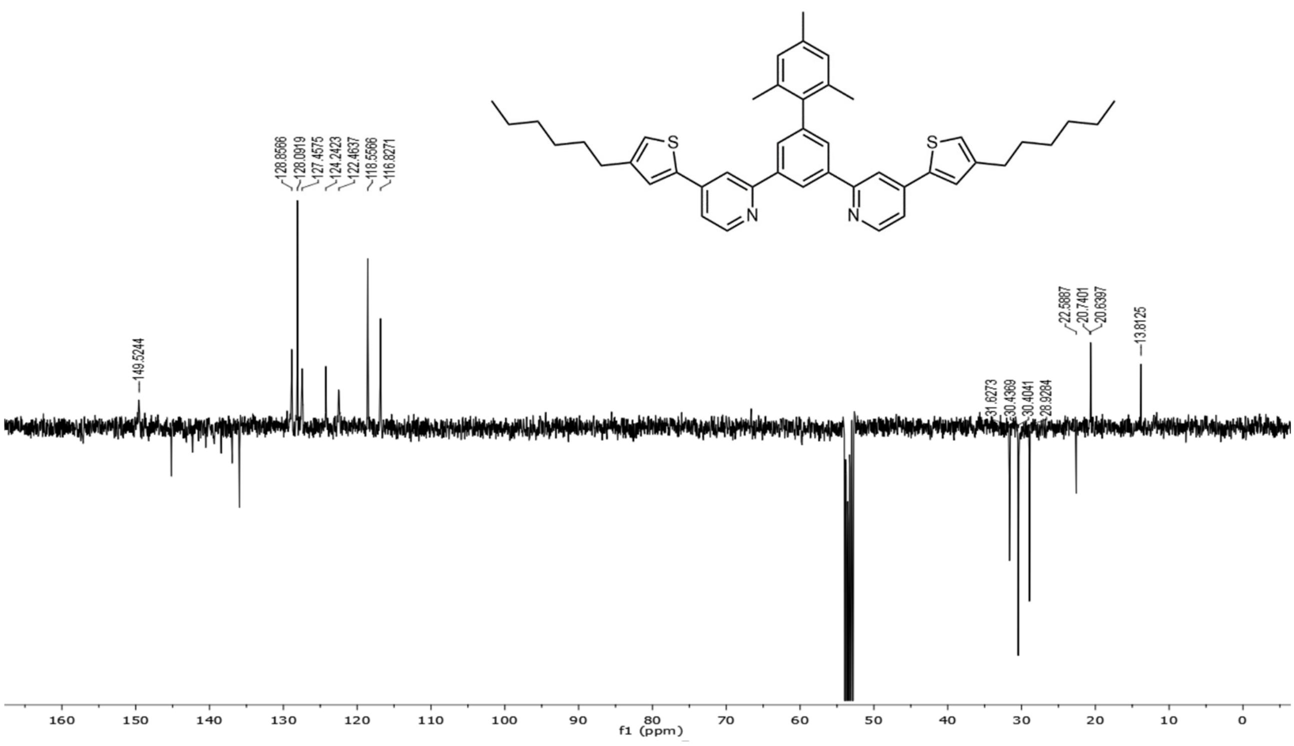

Figure S11. ^{13}C-NMR spectrum of ligand **HL^1**.

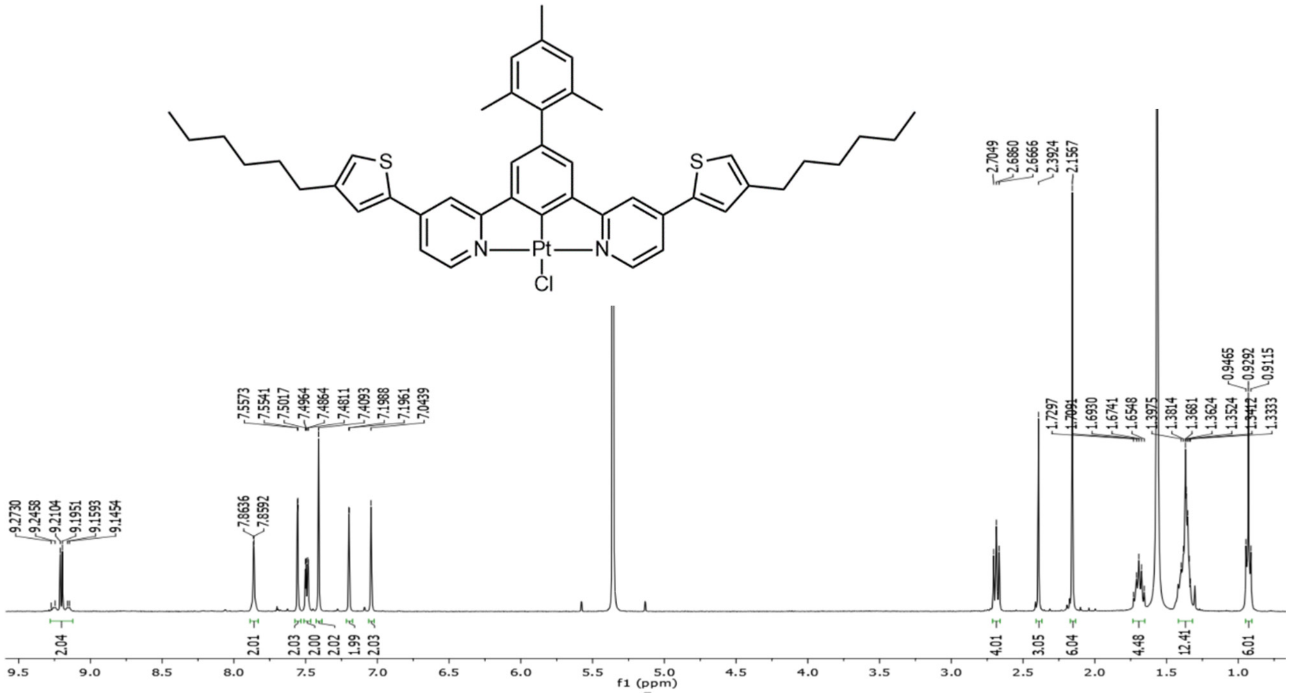

Figure S12. ^{1}H-NMR spectrum of complex **PtL^1Cl**.

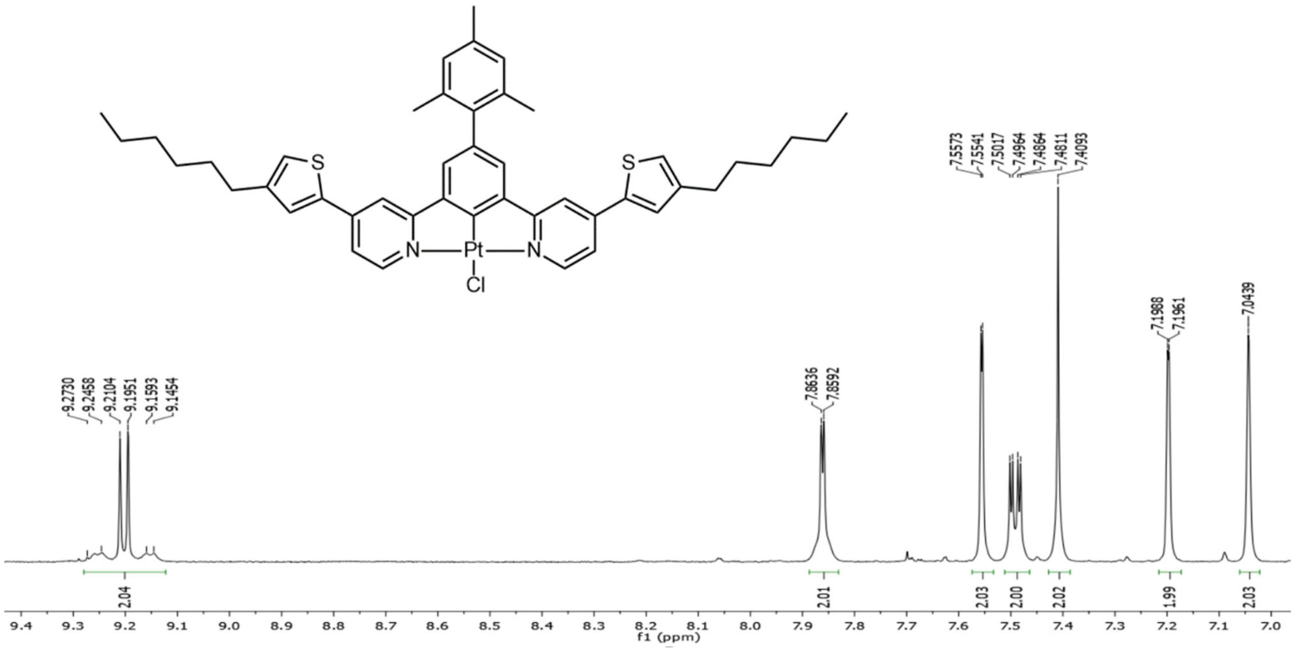

Figure S13. ^{1}H-NMR spectrum of complex **PtL^{1}Cl**, aromatic region.

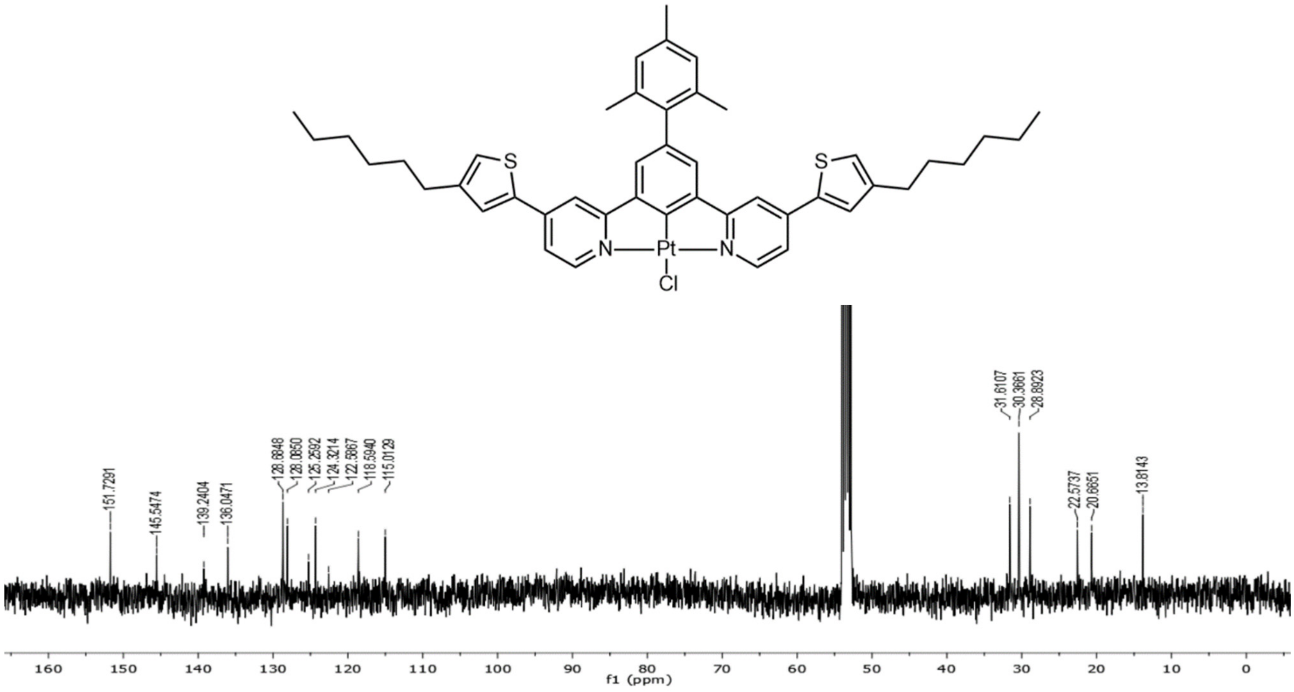

Figure S14. ^{13}C-NMR spectrum of complex **PtL^{1}Cl**.

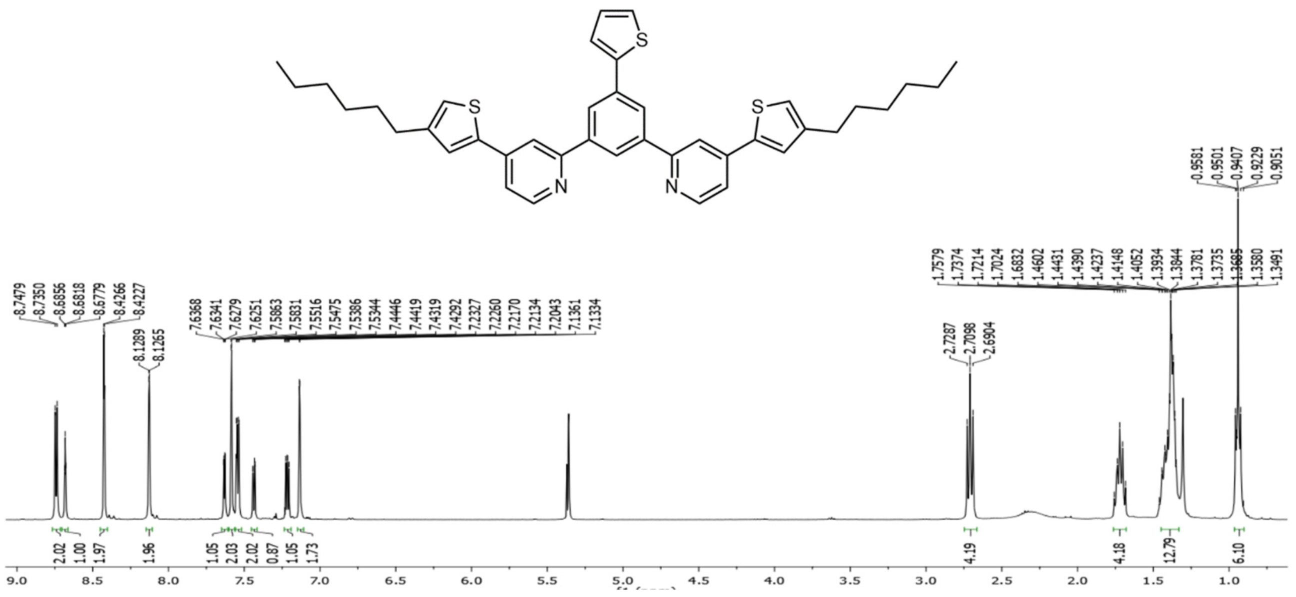

Figure S15. ^{1}H-NMR spectrum of ligand **HL^{2}**.

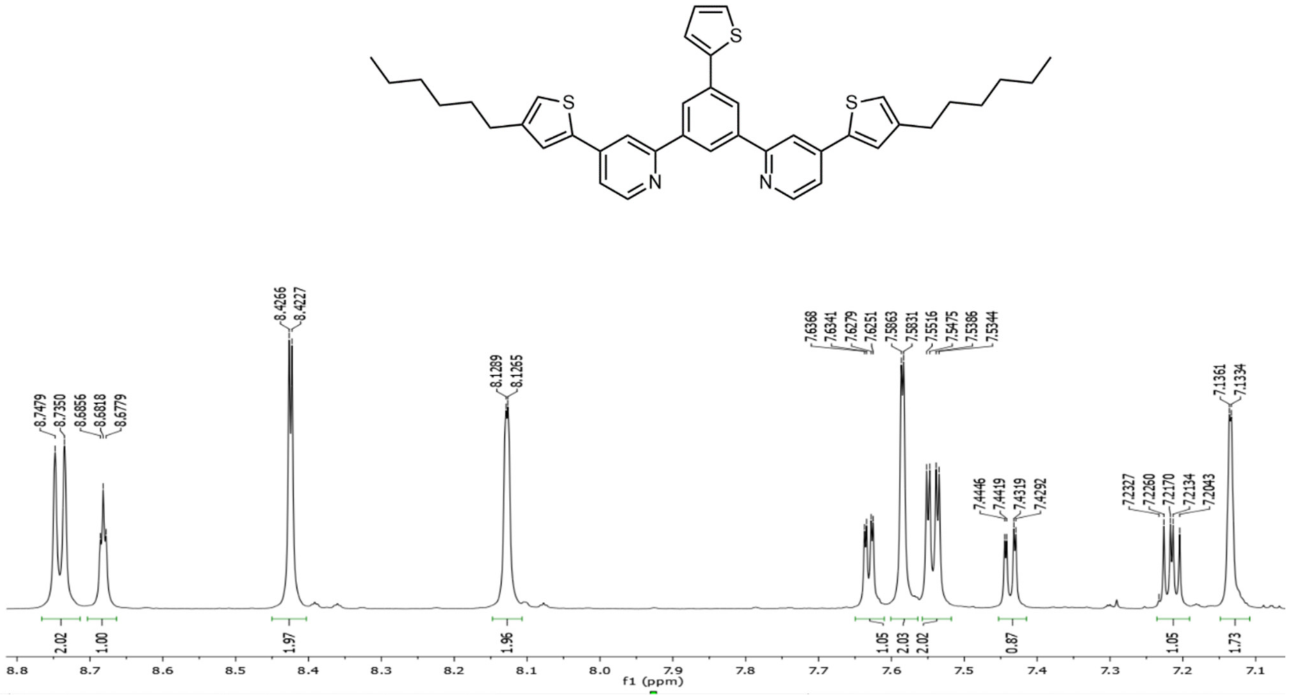

Figure S16. ^{1}H-NMR spectrum of ligand **HL^{2}**, aromatic region.

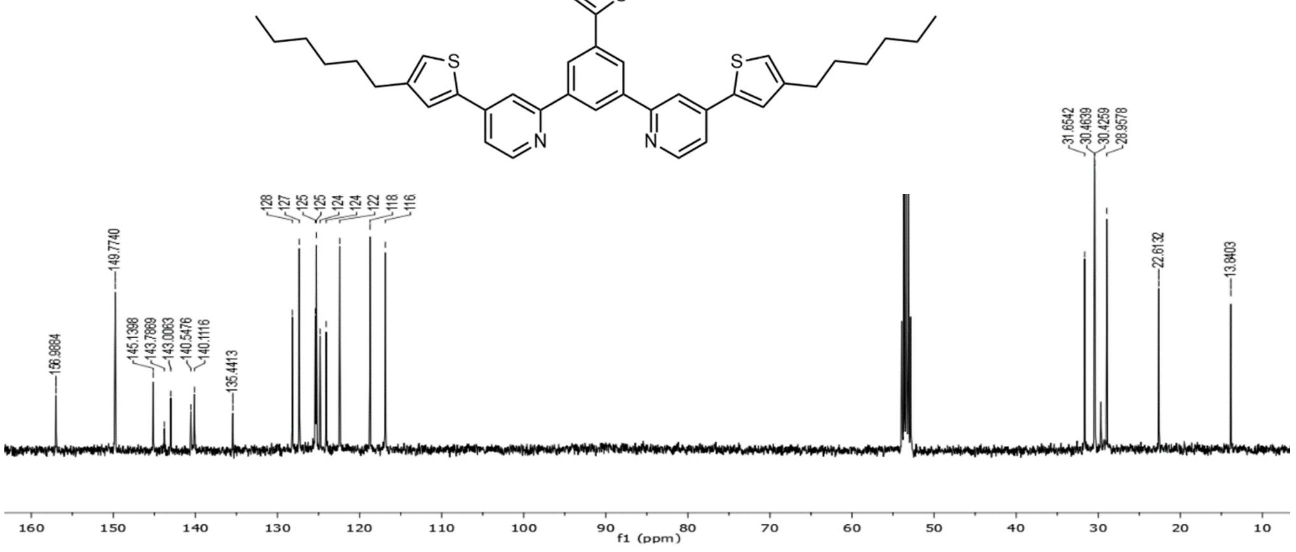

Figure S17. ^{13}C-NMR spectrum of ligand **HL^{2}**.

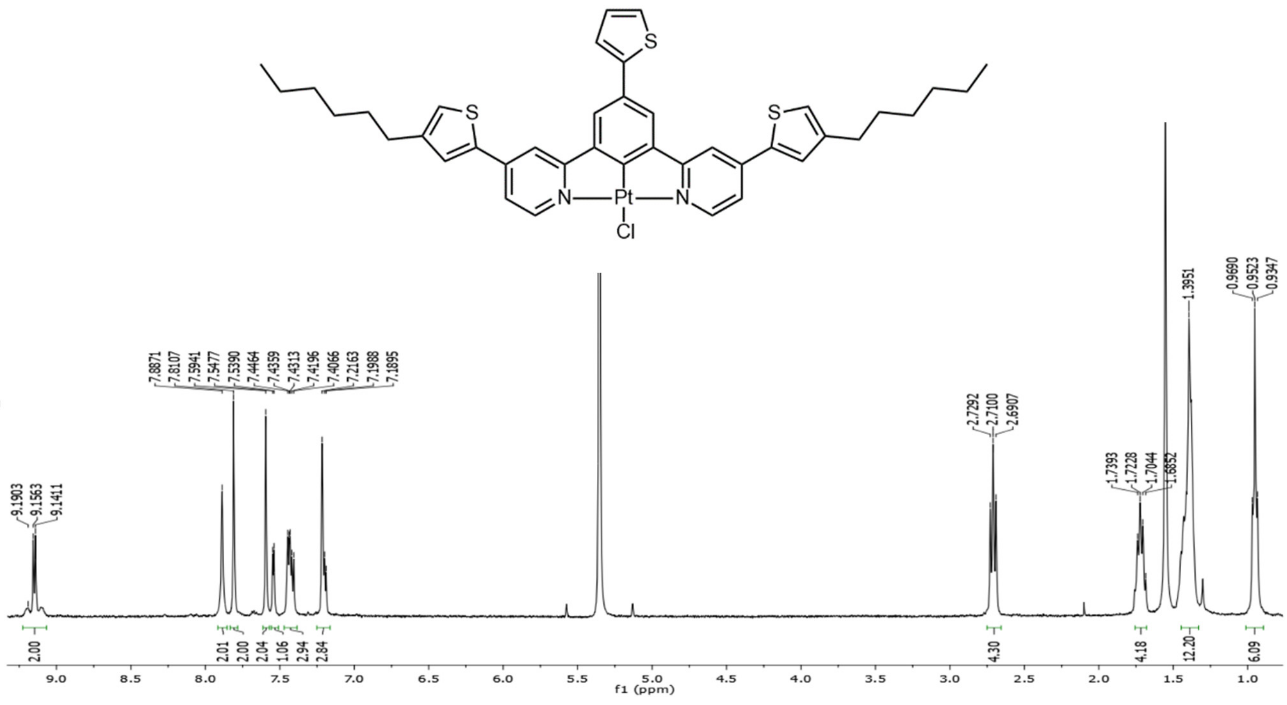

Figure S18. ^{1}H-NMR spectrum of complex **PtL^{2}Cl**.

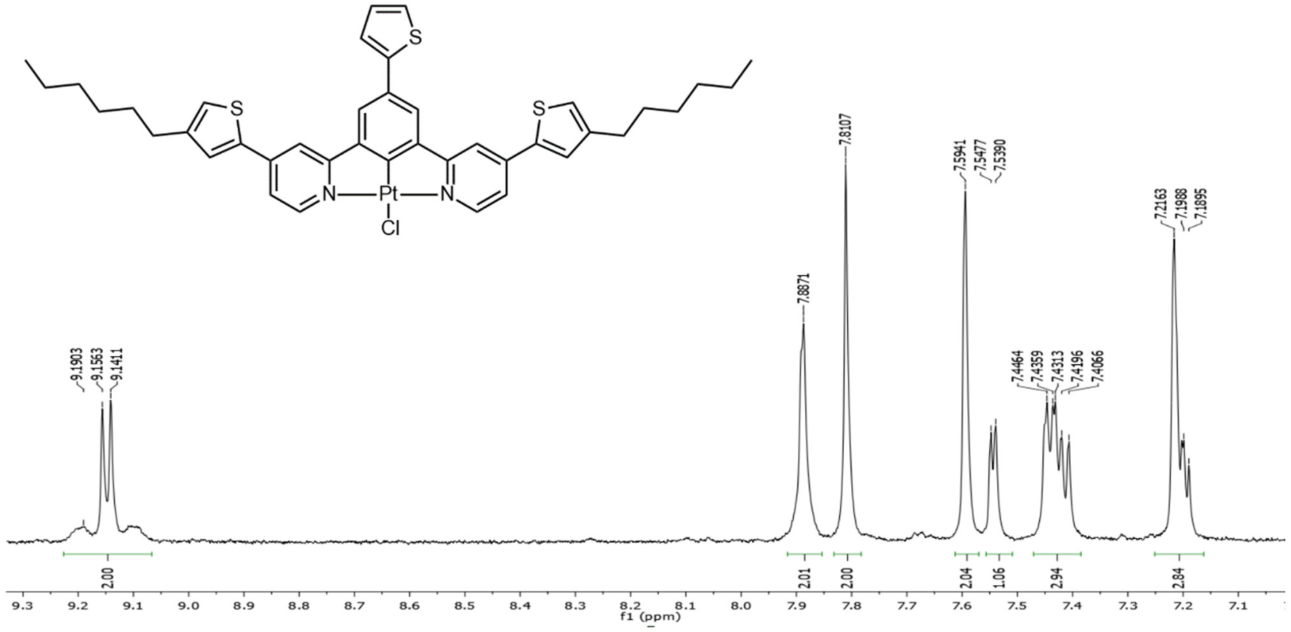

Figure S19. ^{1}H-NMR spectrum of complex **PtL^{2}Cl**, aromatic region.

3. Photophysical characterization of PtL^1Cl and PtL^2Cl in solution

General comments

UV-Visible spectra were collected with a Shimadzu UV3600 spectrophotometer.

Luminescence measurements were carried out in CH_2Cl_2 solution after the Freeze-Pump-Thaw (FPT) procedure, necessary to remove dissolved oxygen.

Absolute photoluminescence quantum yield (Φ) was measured using a C11347 Quantaurus Hamamatsu Photonics K.K spectrometer. A description of the experimental setup and measurement method can be found in the article of K. Suzuki *et al.*[1]

Φ was calculated through Equation:

$$\Phi = \frac{PN\ (Em)}{PN\ (Abs)} = \frac{\int \frac{\lambda}{hc}\left[I_{em}^{sample}(\lambda) - I_{em}^{reference}(\lambda)\right]d\lambda}{\int \frac{\lambda}{hc}\left[I_{exc}^{sample}(\lambda) - I_{exc}^{reference}(\lambda)\right]d\lambda}$$

where PN(Em) is the number of emitted photons, PN(Abs) the number of absorbed photons, λ the wavelength, h the Planck's constant, c the speed of light, I_{em}^{sample} and $I_{em}^{reference}$ the photoluminescence intensities of the sample solution and reference in CH_2Cl_2, I_{exc}^{sample} and $I_{exc}^{reference}$ the excitation light intensities of the sample solution and reference in CH_2Cl_2. PN(Em) is calculated in the wavelength interval [λ_i, λ_f], where λ_i is taken 10 nm below the excitation wavelength, while λ_f is the upper end wavelength in the emission spectrum.

Steady state and time-resolved fluorescence data were obtained using a FLS980 spectrofluorimeter (Edinburg Instrument Ltd). Emission spectra were corrected for background intensity and quantum efficiency of the photomultiplier tube. Excitation spectra were corrected for the intensity fluctuation of a 450 W Xenon arc lamp. Quartz cuvettes with 1 cm optical path length were used.

Time-resolved fluorescence measurements were performed through the time-correlated single photon counting technique with an Edinburgh Picosecond Pulsed Diode Laser (emitted wavelength 374 nm).

Moreover, time-resolved fluorescence curves were fitted using an exponential function:

$$I(\lambda, t) = \alpha(\lambda) \exp\left(\frac{-t}{\tau}\right)$$

where α (λ) is the amplitude at wavelength λ and τ is the lifetime. The quality of the fit was evaluated through the reduced χ^2 values.

In case of multi-exponential decay, the average lifetime (τ_{av}) was calculated as follows:

$$\tau_{av} = \frac{\sum_{n=1}^{m} \alpha_n \tau_n^2}{\sum_{n=1}^{m} \alpha_n \tau_n}$$

where *m* is the multi-exponential decay number of the fit.

Absorption spectra and molar extinction coefficients

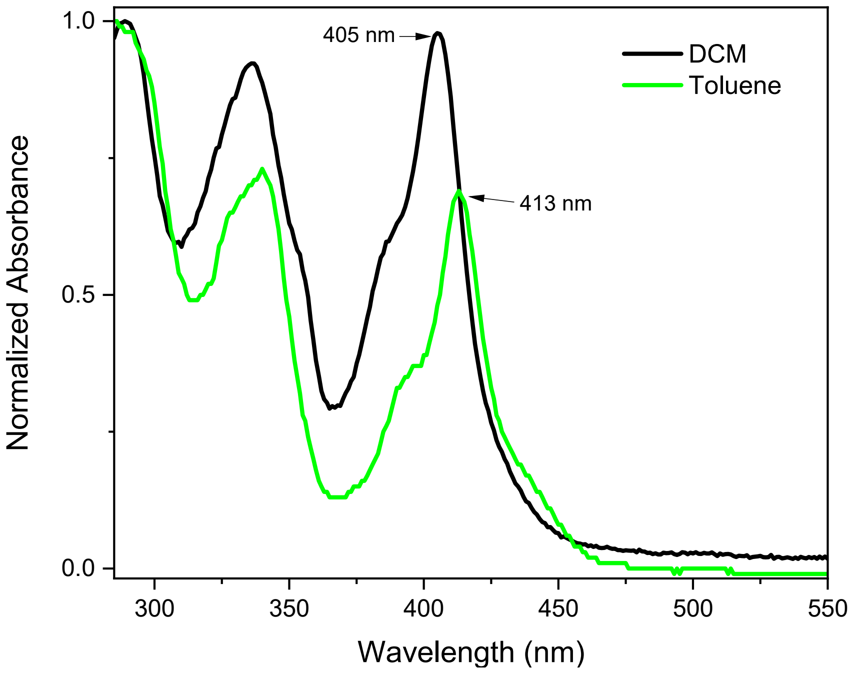

Figure S20. Normalized absorption spectra of dilute ($2 \cdot 10^{-6}$ M) solutions of **PtL^{1}Cl** in dichloromethane and in toluene.

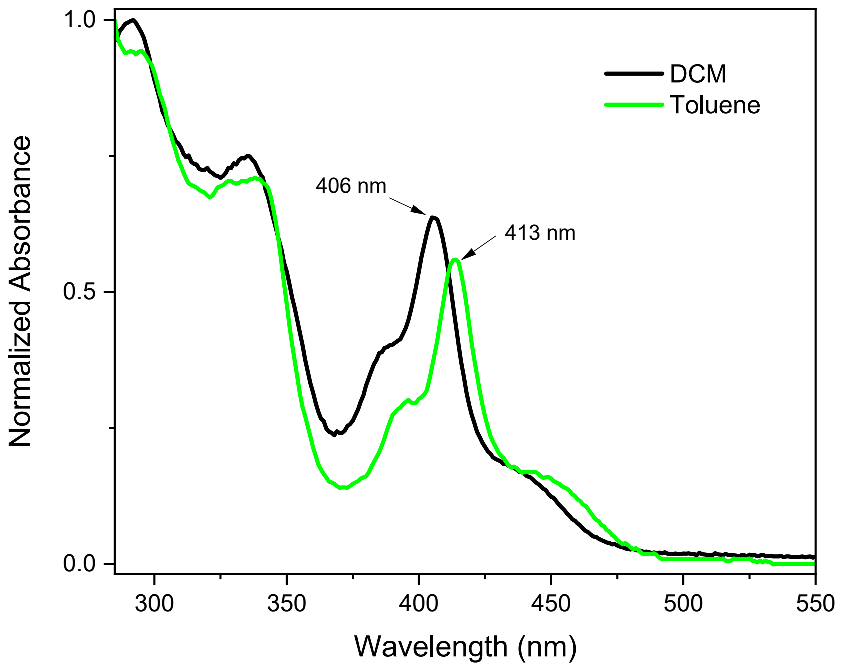

Figure S21. Normalized absorption spectra of dilute ($2 \cdot 10^{-6}$ M) solutions of **PtL^{2}Cl** in dichloromethane and in toluene.

Table S1. Absorption maxima and molar extinction coefficients for complexes **PtL^{1}Cl** and **PtL^{2}Cl** in dichloromethane.

**Complex**		$\lambda_{max,\ abs}$ / nm	ε / (10^3 cm^{-1} M^{-1})
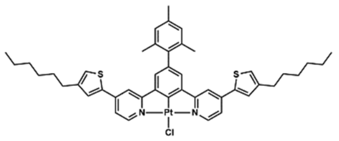	**PtL^{1}Cl**	289<br>336<br>405	43.6<br>40.2<br>43.1
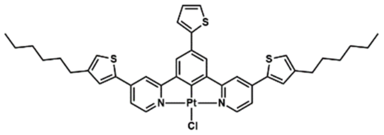	**PtL^{2}Cl**	291<br>335<br>406	53.6<br>41.0<br>34.8

Excitation and Emission spectra

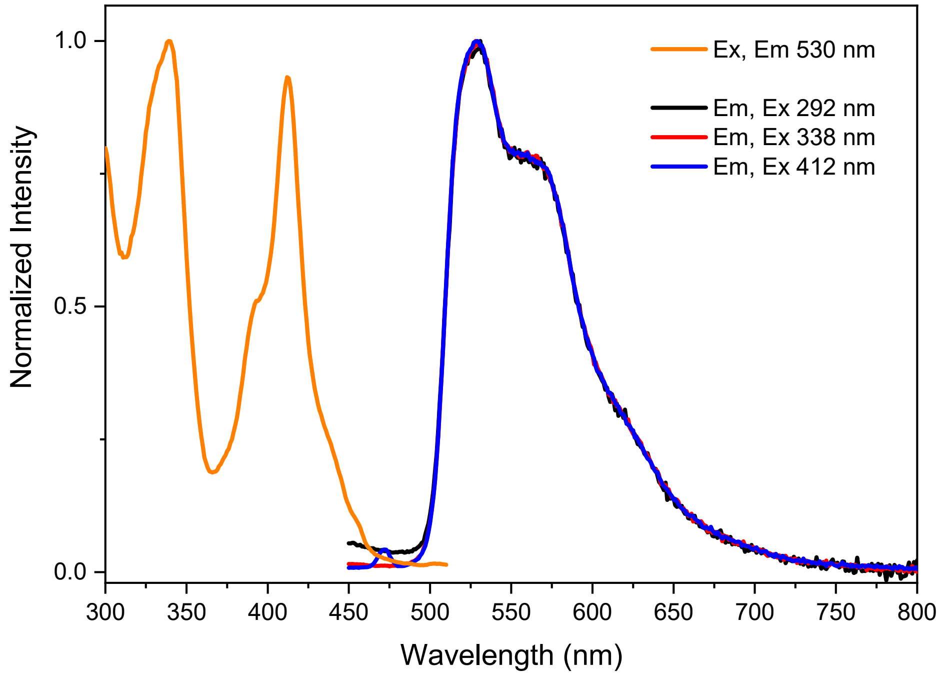

Figure S22. Normalized excitation and emission spectra of a dilute ($2\cdot10^{-6}$ M) solution of **PtL^{1}Cl** in toluene.

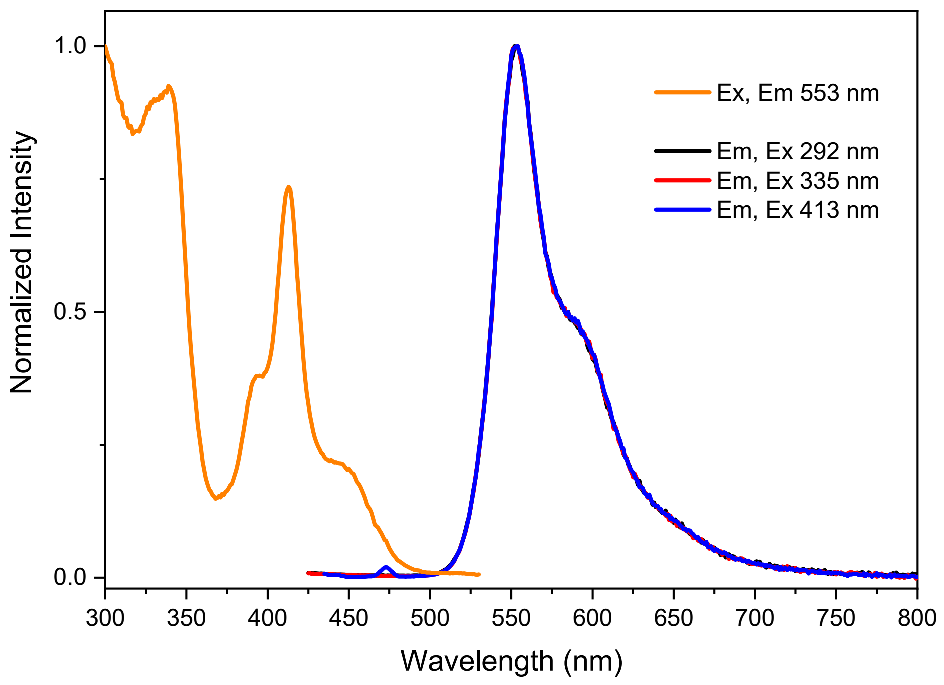

Figure S23. Normalized excitation and emission spectra of a dilute ($2\cdot10^{-6}$ M) solution of **PtL²Cl** in toluene.

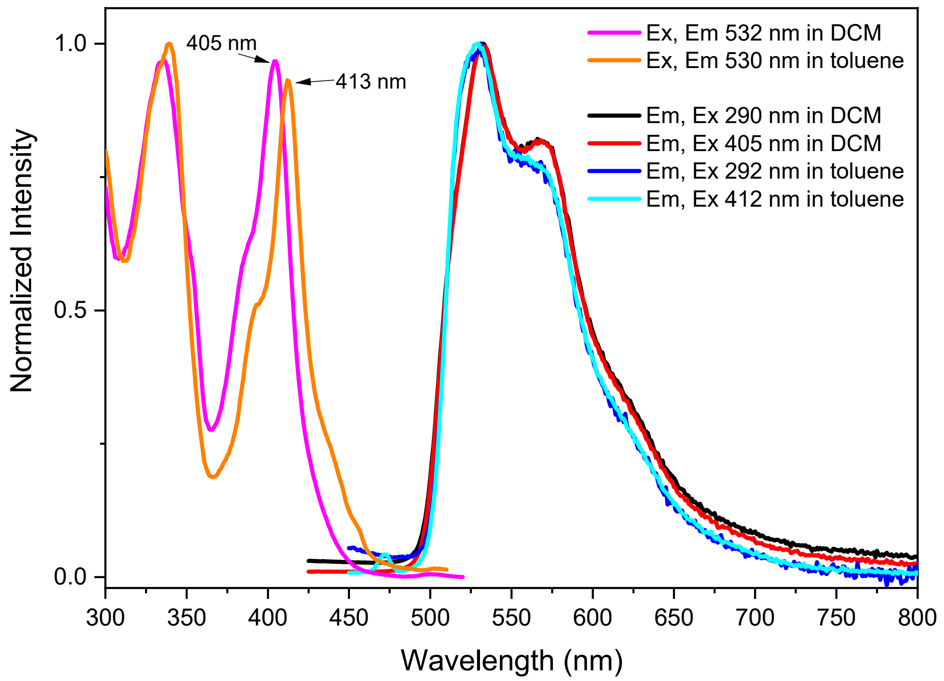

Figure S24. Comparison between the normalized excitation and emission spectra of dilute ($2\cdot10^{-6}$ M) solutions of **PtL¹Cl** in dichloromethane and in toluene.

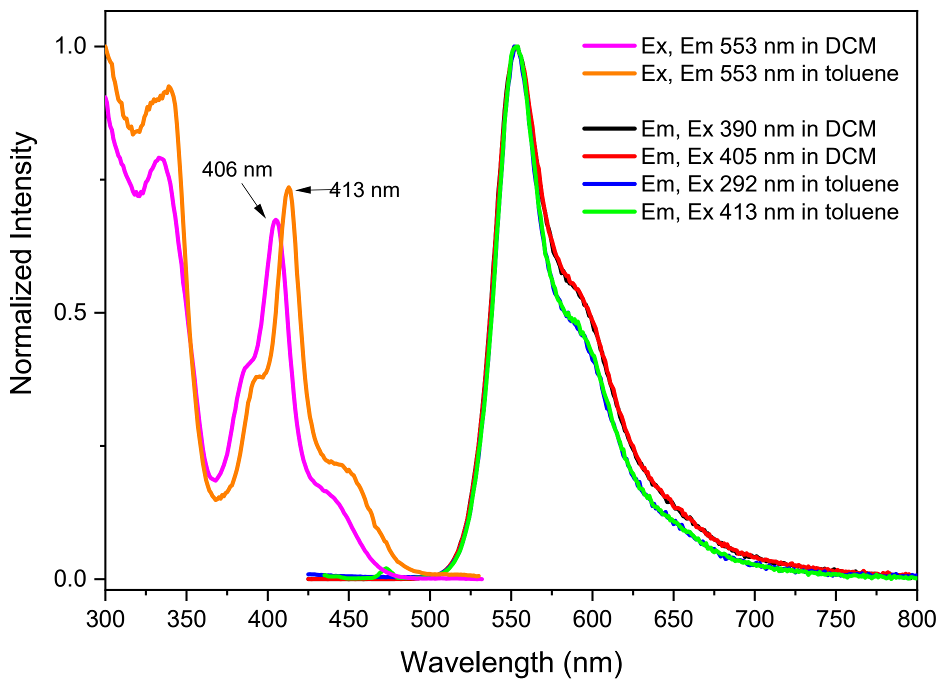

Figure S25. Comparison between the normalized excitation and emission spectra of dilute ($2\cdot10^{-6}$ M) solutions of **PtL^{2}Cl** in dichloromethane and in toluene.

Absolute Quantum Yields and lifetimes

Table S2. Absolute Quantum Yields and lifetimes for complexes **PtL^{1}Cl** and **PtL^{2}Cl**.

Complex		c / M	QY_{after} (QY_{before})	τ / µs	λ_{ex} , λ_{em} / nm
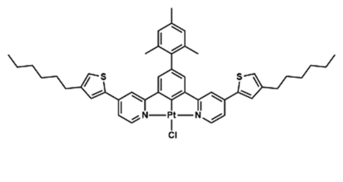	**PtL^{1}Cl**	$2\cdot10^{-6}$	96.4% (2.6%)	24.08	405, 532
				23.73	405, 725
		$2\cdot10^{-4}$	50.1% (2.1%)	6.06	405, 532
				6.29	405, 753
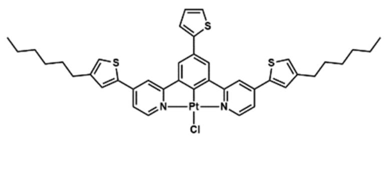	**PtL^{2}Cl**	$2\cdot10^{-6}$	99.0% (6.0%)	5.94	405, 553
				6.01	405, 700
		$2\cdot10^{-4}$	42.5% (4.6%)	1.41	405, 553
				1.51	405, 740

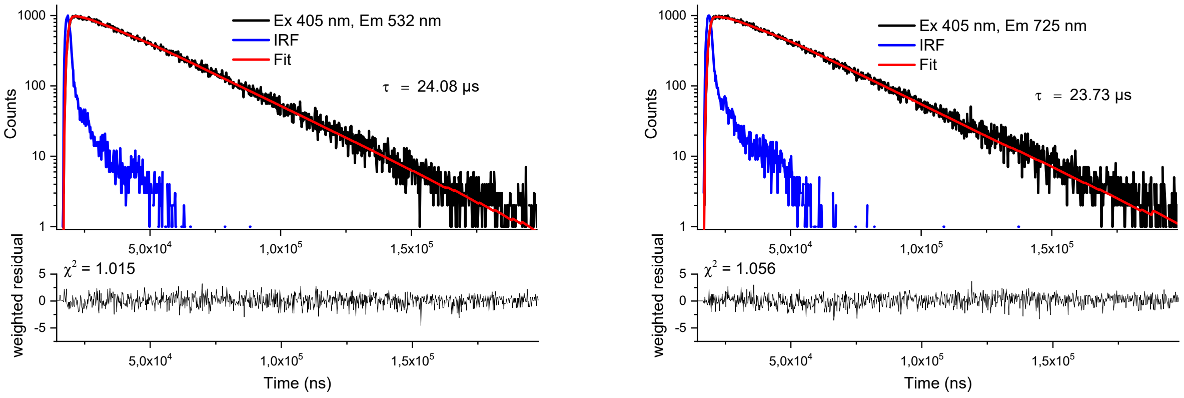

Figure S26. Lifetime measurements of a dilute ($2 \cdot 10^{-6}$ M) dichloromethane solution of **PtL^{1}Cl**.

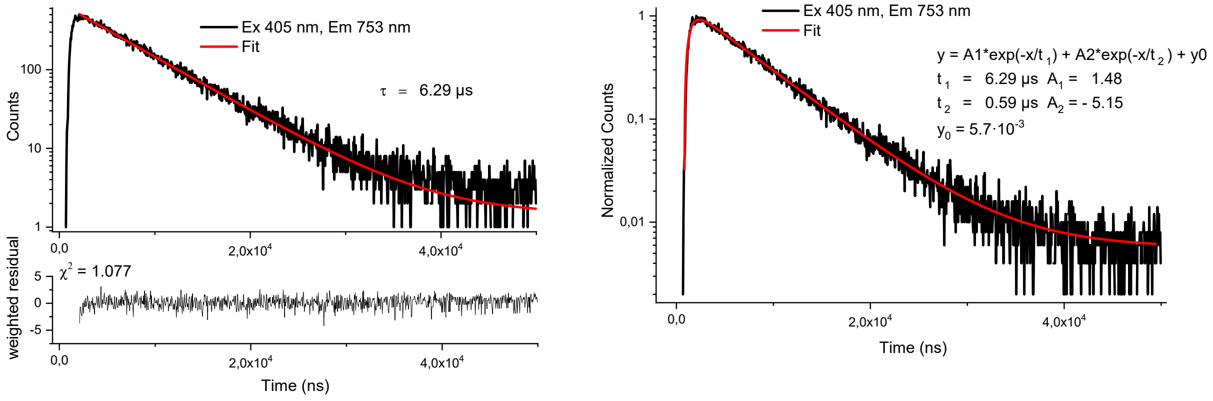

Figure S27. Lifetime measurements of a concentrated ($2 \cdot 10^{-4}$ M) dichloromethane solution of **PtL^{1}Cl**.

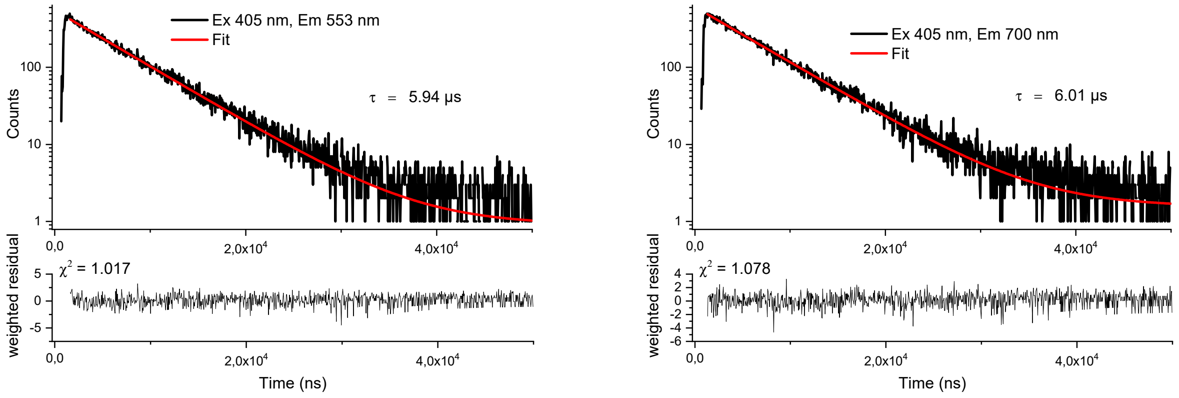

Figure S28. Lifetime measurements of a dilute ($2 \cdot 10^{-6}$ M) dichloromethane solution of **PtL²Cl**.

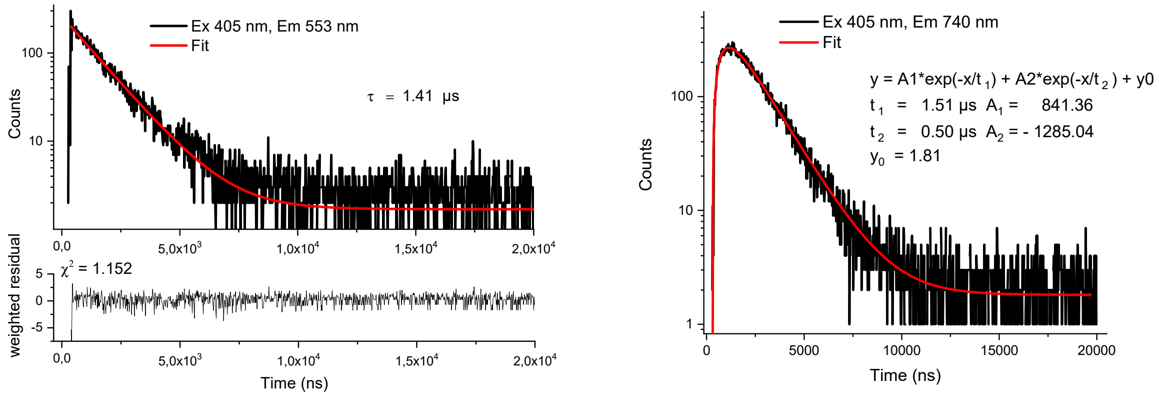

Figure S29. Lifetime measurements of a concentrated ($2 \cdot 10^{-4}$ M) dichloromethane solution of **PtL²Cl**.

4. Photophysical characterization of PtL¹Cl and PtL²Cl in thin films

General comments

Thin films containing 1 wt% of complexes in poly-methylmethacrylate (PMMA, Mw ≈ 15000 g/ mol) on quartz plate (thickness 1 mm) were obtained by spin-coating (Cookson Electronic Company P-6708D). The parameters of spinning (RPM-revolutions per minute) were RPM 1: 800; Ramp 1: 1 s, Time 1: 5 s; RPM 2: 2000; Ramp 2: 1 s, Time 2: 120 s; RPM 3: 4000; Ramp 3: 2 s, Time 30 s. The solutions for the 1 wt% thin films were prepared with 1.33 mg of complex and 133 mg of PMMA in 1 ml of dichloromethane.

Absorption spectra

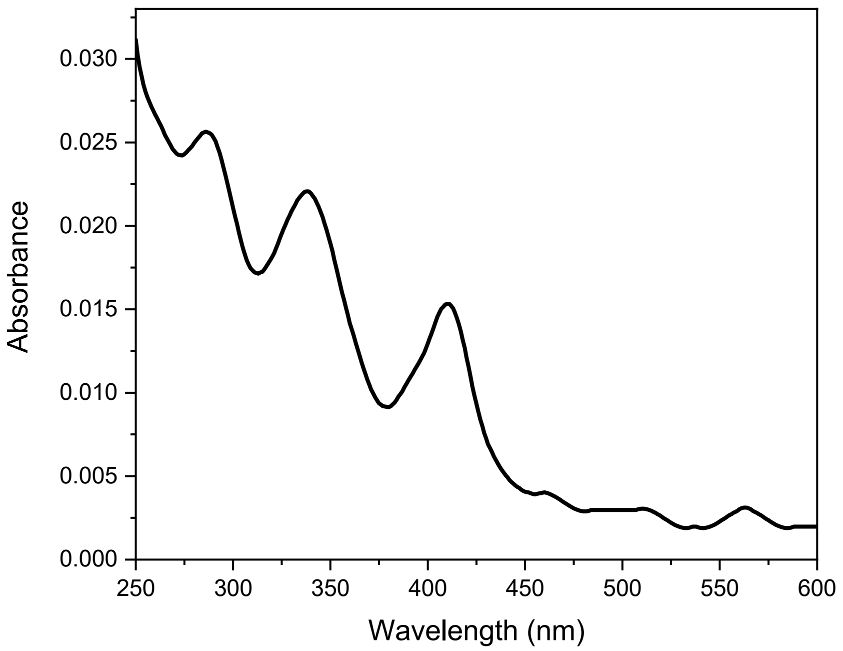

Figure S30. Absorption spectrum of a 1% w/w film of **PtL¹Cl** in PMMA.

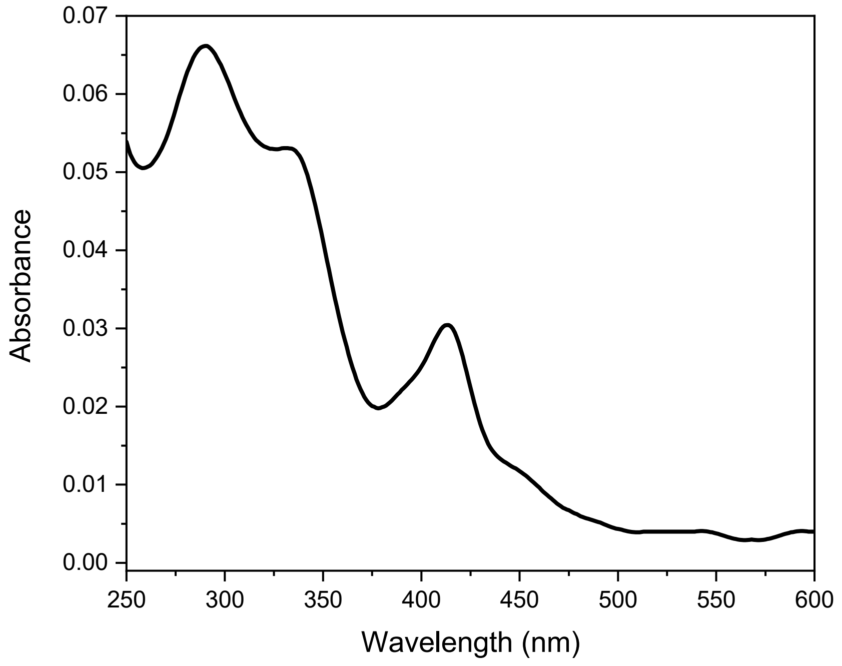

Figure S31. Absorption spectrum of a 1% w/w film of **PtL^{2}Cl** in PMMA.

Excitation and Emission spectra

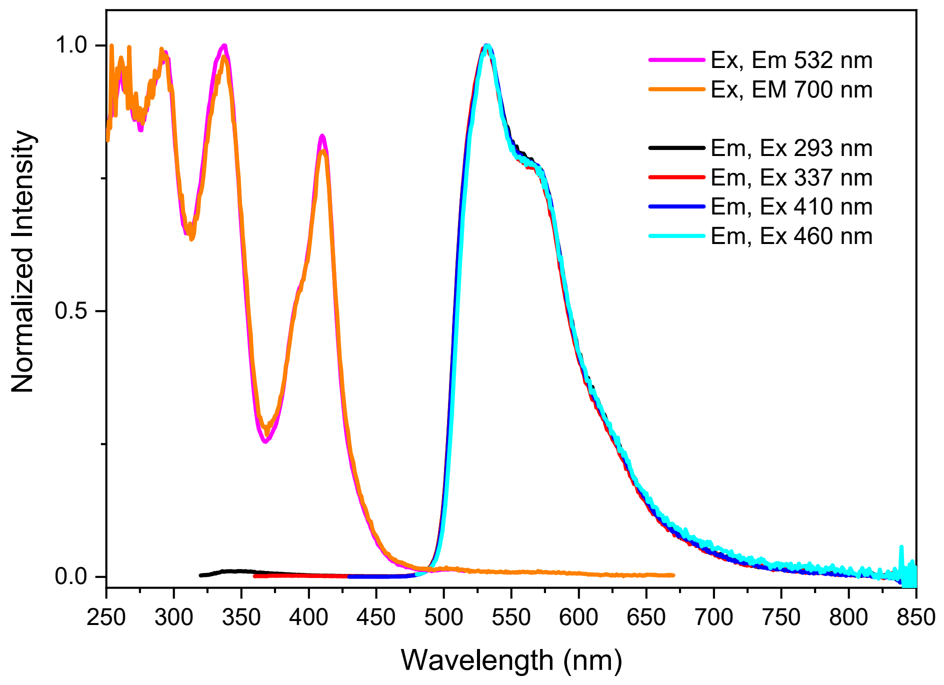

Figure S32. Excitation and Emission spectra of a 1% w/w film of **PtL^{1}Cl** in PMMA.

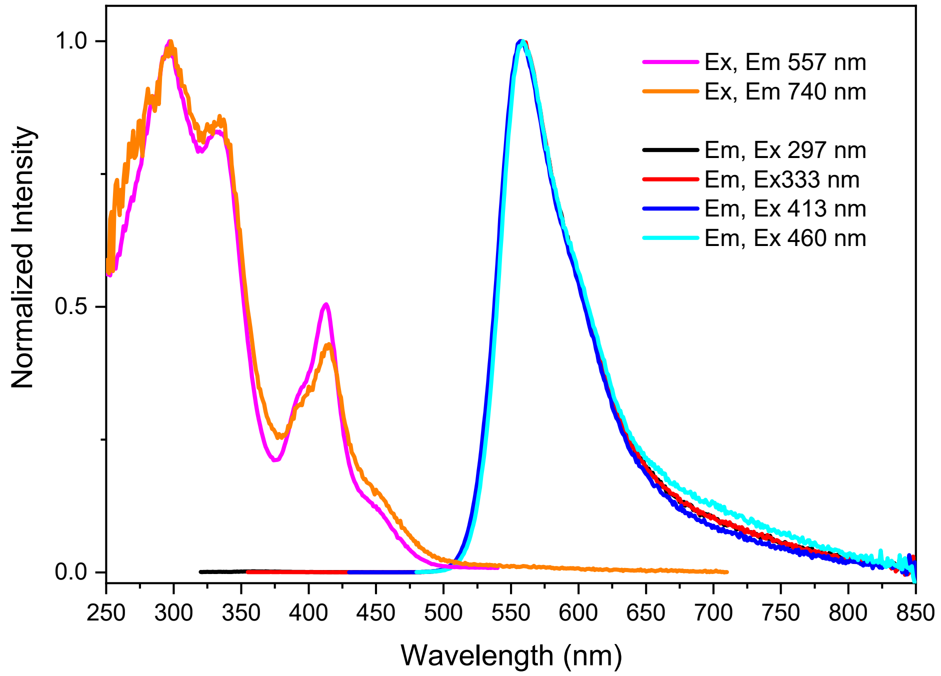

Figure S33. Excitation and Emission spectra of a 1% w/w film of **PtL²Cl** in PMMA.

Absolute Quantum Yields and lifetimes

Table S3. Absolute Quantum Yields and lifetimes of 1% w/w films of **PtL¹Cl** and **PtL²Cl** in PMMA.

Complex		QY_{after} (QY_{before})	τ / μs	λ_{ex} , λ_{em} / nm	k_r / s^{-1}	k_{nr} / s^{-1}
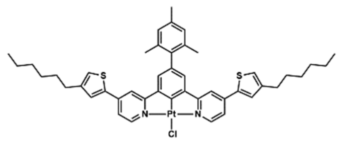	**PtL¹Cl**	49.7%	16.42	405, 532	$3.03 \cdot 10^4$	$3.06 \cdot 10^4$
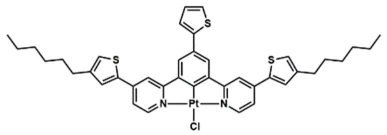	**PtL²Cl**	39.6%	5.09	405, 557	$7.78 \cdot 10^4$	$1.19 \cdot 10^5$

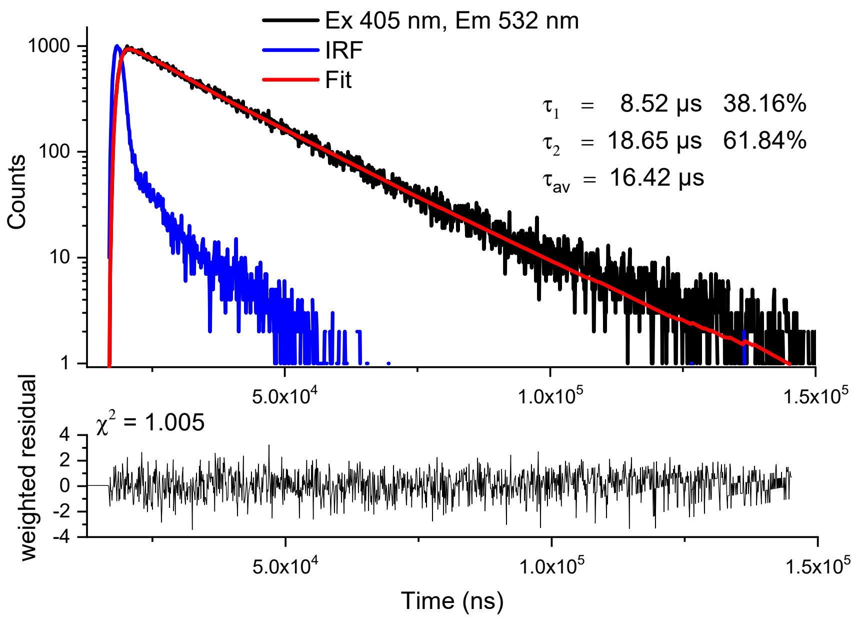

Figure S34. Lifetime measurements of a 1% w/w film of **PtL^{1}Cl** in PMMA.

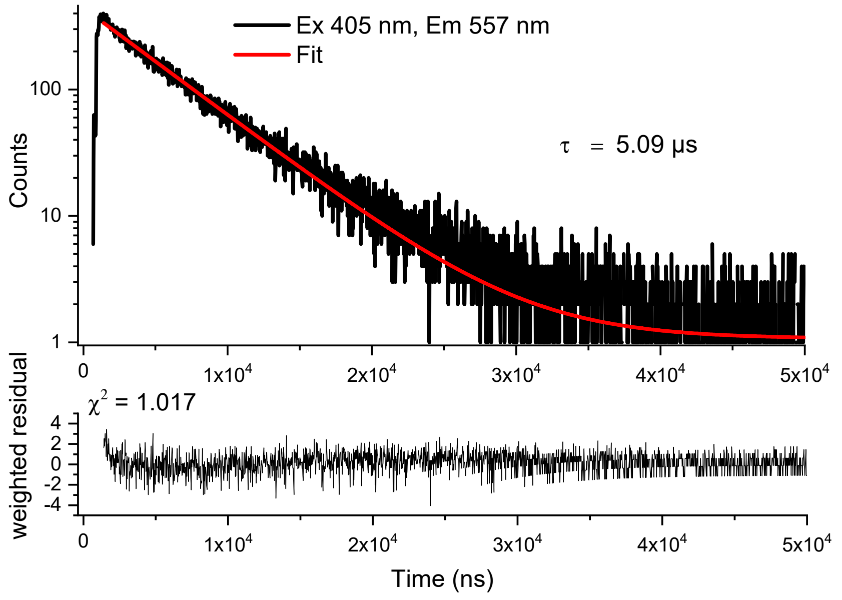

Figure S35. Lifetime measurements of a 1% w/w film of **PtL^{2}Cl** in PMMA.

5. Theoretical calculations

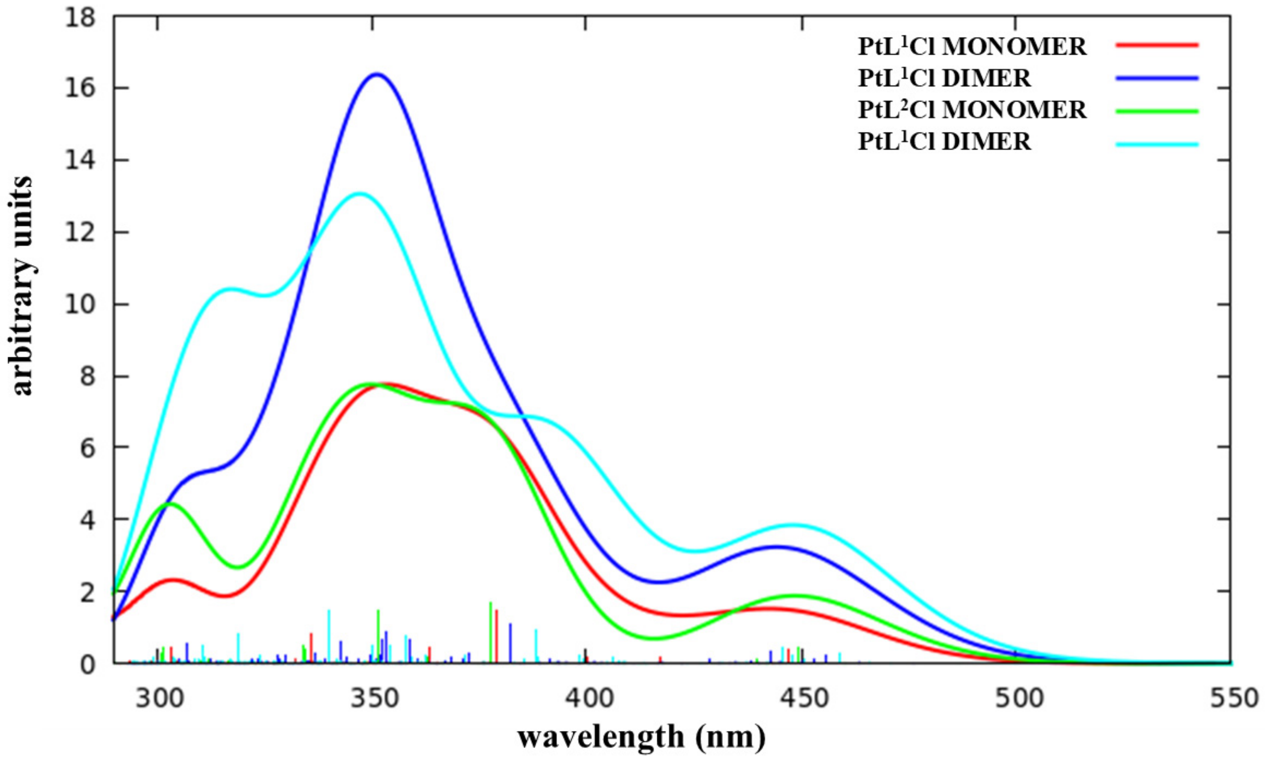

Figure S36. Simulated absorption spectra of **PtL^{1}Cl** and **PtL^{2}Cl** monomers and dimers.

6. References

1. Suzuki, K.; Kobayashi, A.; Kaneko, S.; Takehira, K.; Yoshihara, T.; Ishida, H.; Shiina, Y.; Oishic, S.; Tobita, S. *Phys. Chem. Chem. Phys.*, 2009, 11, 9850–9860.
